# Hemodynamic performance of tissue-engineered vascular grafts in Fontan patients

**DOI:** 10.1038/s41536-021-00148-w

**Published:** 2021-07-22

**Authors:** Erica L. Schwarz, John M. Kelly, Kevin M. Blum, Kan N. Hor, Andrew R. Yates, Jacob C. Zbinden, Aekaansh Verma, Stephanie E. Lindsey, Abhay B. Ramachandra, Jason M. Szafron, Jay D. Humphrey, Toshiharu Shin’oka, Alison L. Marsden, Christopher K. Breuer

**Affiliations:** 1 Department of Bioengineering, Stanford University, Stanford, CA USA; 2 Center for Regenerative Medicine, Abigail Wexner Research Institute at Nationwide Children’s Hospital, Columbus, OH USA; 3 The Heart Center, Nationwide Children’s Hospital, Columbus, OH USA; 4 Department of Biomedical Engineering, The Ohio State University, Columbus, OH USA; 5 Department of Pediatrics, The Ohio State University College of Medicine, Columbus, OH USA; 6 Department of Pediatrics, Stanford University, Stanford, CA USA; 7 Department of Biomedical Engineering, Yale University, New Haven, CT USA; 8 Department of Cardiothoracic Surgery, Nationwide Children’s Hospital, Columbus, OH USA; 9 Department of Surgery, The Ohio State University Wexner Medical Center, Columbus, OH USA; 10 Department of Surgery, Nationwide Children’s Hospital, Columbus, OH USA

**Keywords:** Tissue engineering, Circulation, Translational research

## Abstract

In the field of congenital heart surgery, tissue-engineered vascular grafts (TEVGs) are a promising alternative to traditionally used synthetic grafts. Our group has pioneered the use of TEVGs as a conduit between the inferior vena cava and the pulmonary arteries in the Fontan operation. The natural history of graft remodeling and its effect on hemodynamic performance has not been well characterized. In this study, we provide a detailed analysis of the first U.S. clinical trial evaluating TEVGs in the treatment of congenital heart disease. We show two distinct phases of graft remodeling: an early phase distinguished by rapid changes in graft geometry and a second phase of sustained growth and decreased graft stiffness. Using clinically informed and patient-specific computational fluid dynamics (CFD) simulations, we demonstrate how changes to TEVG geometry, thickness, and stiffness affect patient hemodynamics. We show that metrics of patient hemodynamics remain within normal ranges despite clinically observed levels of graft narrowing. These insights strengthen the continued clinical evaluation of this technology while supporting recent indications that reversible graft narrowing can be well tolerated, thus suggesting caution before intervening clinically.

## Introduction

Improved biomaterials promise to reduce the morbidity and mortality associated with congenital heart disease. The common lack of homologous tissue for reconstructive surgery necessitates the use of cadaveric, xenograft, or synthetic materials^1^. These biomaterials have limited durability and, particularly relevant to the pediatric population, they lack growth potential^2^. Their use is associated with the need for repeat operations secondary to somatic overgrowth or deterioration of graft function^3^. Tissue engineering offers an alternative to currently available biomaterials by stimulating the growth of autologous tissue for surgical repair, replacement, or reconstruction^4^. The general approach involves harvesting human cells that are then seeded onto a scaffold. Replacement tissue can be generated through either in vitro culture or by inducing autologous tissue regeneration in vivo^5^.

Our group pioneered the use of tissue-engineered vascular grafts (TEVGs) created by seeding autologous bone marrow-derived mononuclear cells onto a tubular biodegradable polymer scaffold^6^. This scaffold is implanted directly, and over time, it degrades as the TEVG develops into a neovessel that has the ability to grow and remodel^7,8^. This graft technology has been applied to the modified extracardiac Fontan procedure for single ventricle palliation whereby the inferior vena cava (IVC) is connected to the pulmonary arteries via a vascular graft. The Fontan circulation, with its high volumetric flow rate and low pressure conditions, was purposely selected to evaluate the safety and efficacy of this graft construct given the relatively low risk of catastrophic complications such as acute thrombosis or graft dilation and rupture within a large caliber vessel at low pressure.

Poly(tetrafluoroethylene) (ePTFE), a synthetic polymer, is the traditional material of choice for the extracardiac Fontan conduit given a lack of appropriate autologous tissue for constructing a large caliber blood vessel. Yet, ePTFE grafts engender several known risk factors including stenosis, thrombosis, and calcification, with the additional disadvantage of lack of growth potential^9–12^. The current standard of care for single ventricle palliation is a three-stage approach, based on clinical experience of improved survival following the introduction of the modified bidirectional Glenn procedure (superior vena cava (SVC) anastomosed to the right pulmonary artery (RPA)) as an interim palliation prior to Fontan completion^13^. Optimal timing for staged single ventricle palliation remains controversial, but the lack of growth potential of synthetic grafts necessitates delayed insertion and oversizing to ensure long-term functionality. Delayed insertion subjects the patient to a period of progressively worsening cyanosis and hypoxia, which may affect patient development, whereas oversized grafts may affect the hemodynamic performance of the Fontan circuit until the child grows^14–18^.

Clinical application of our TEVG technology in Fontan patients has shown that these grafts can be successfully implanted without significant long-term risk of thrombosis, calcification, or aneurysmal dilation^7,8,16,19,20^. The primary graft-related complication has been stenosis, or narrowing, of the graft^20^. While the majority of patients with graft stenosis were asymptomatic and the stenosis was successfully treated with balloon angioplasty, stenosis represents a major hurdle to widespread clinical adoption of this otherwise promising technology. Understanding the natural history of TEVG remodeling within the Fontan circulation and how these changes impact hemodynamic performance is critical to the continued use of these grafts. In this study, we provide insight into TEVG behavior in vivo through a detailed analysis of the U.S. clinical trial data. We demonstrate a dynamic period of graft remodeling starting with dilation, then narrowing followed by subsequent growth. Imaging studies confirm a change in structural stiffness from a relatively stiff polymeric scaffold to a more distensible vascular graft. Then, using a computational fluid dynamics (CFD) framework, we investigate how patient-specific changes to conduit geometry and properties affect Fontan hemodynamic performance.

## Results

### Scaffold design and chemical characterization

**Fig. 1:**
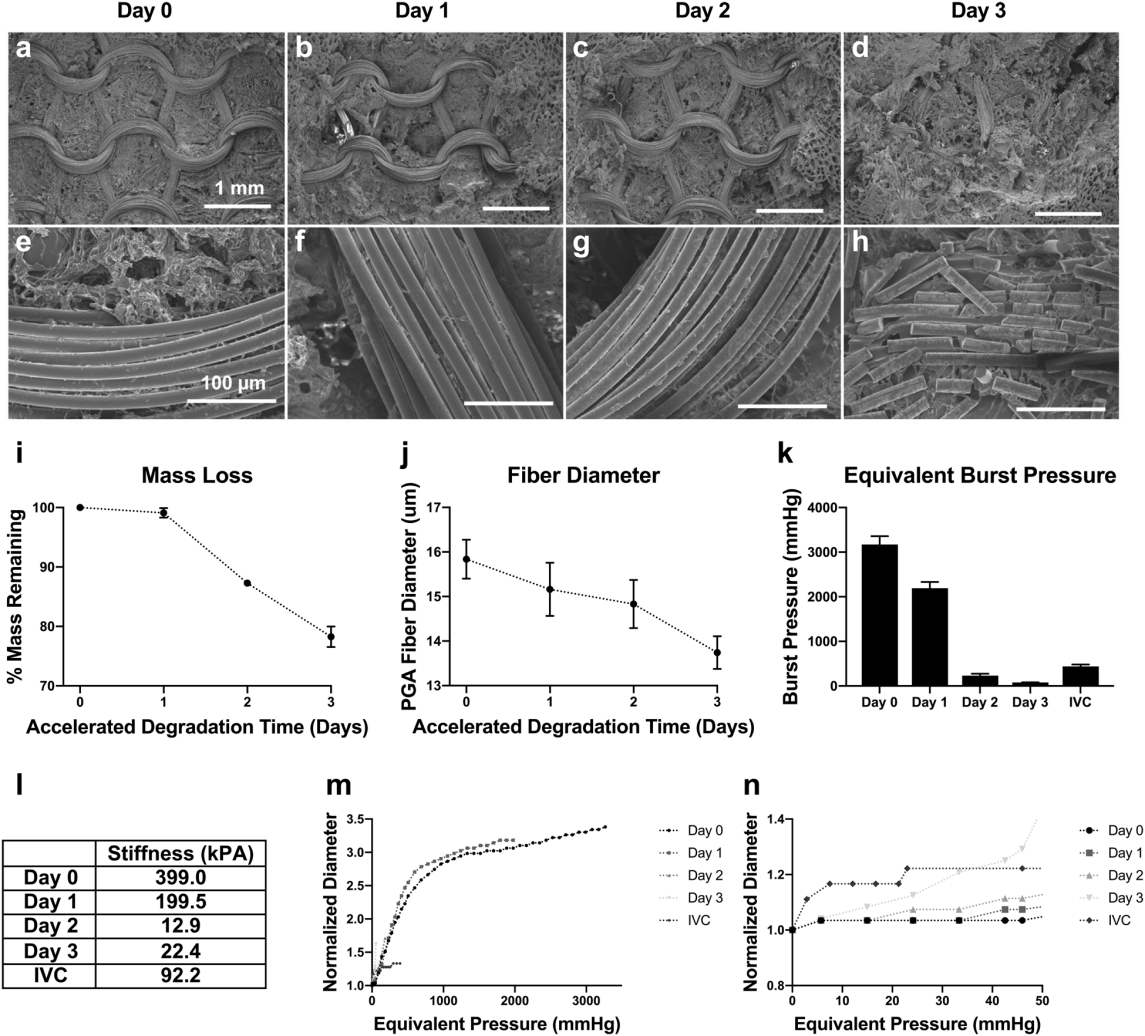
Accelerated in vitro degradation of scaffold. Degradation in heated 1× PBS of TEVG scaffolds to simulate 1 month in vivo with 1 day in vitro. **a**–**d** ×30 and **e**–**h** ×400 scanning electron microscopic (SEM) images of the scaffold throughout degradation, with luminal PCLA layer removed with forceps to expose PGA weave. **i** Dry mass weights of TEVG throughout degradation. **j** Diameter of PGA fibers throughout degradation as measured by FIJI of SEM images (*n* = 5 fibers). **k** Equivalent burst pressure. **l** Calculated scaffold stiffness throughout degradation compared to the measured stiffness of a sheep thoracic IVC. **m** Distensibility testing of TEVG throughout degradation. **n** Distensibility testing data zoomed in on the low, physiologically relevant pressures, including IVC data. (*n* = 3), error bars represent standard deviation.

The scaffold consisted of a middle layer of poly(glycolic acid) (PGA) fibers that were knitted into a tube and then coated on both luminal and abluminal surfaces by a thick porous poly(caprolactone/lactic acid) (PCLA) sponge layers. The PGA fiber bundles were knit in a weft pattern, with 1 mm between layers axially along the TEVG and 1 mm between peaks running circumferentially (Fig. 1a, e). The rapidly degrading PGA fibers provided the main strength to the TEVG at implant, while the longer lasting PCLA mesh provided space for cellular infiltration and matrix deposition.

### Scaffold degradation

The scaffold was designed to degrade by hydrolysis over the first 6 months after implantation^6^. We performed an accelerated degradation study in vitro by submerging scaffold samples in heated 1× phosphate-buffered saline (PBS) solution at 70 °C. Dry weight showed little change within the first day of degradation (1 month equivalent at in vivo temperature), with more rapid degradation occurring at days 2 and 3 (2 and 3 months in vivo temperature; Fig. 1i). Scanning electron microscopic (SEM) assessment of the samples showed that PGA fiber diameter decreased nearly linearly throughout degradation (Fig. 1j). The fibers within the PGA knitted structure showed minor pitting at day 1, more substantial surface pitting and the initial evidence of fracturing at day 2, and substantial breaking of the knitted structure by day 3. As the in vivo degradation of the TEVG is complicated by cellular invasion, neotissue deposition, fluid flow, and local pH differences, it is important to note that this in vitro testing serves as an estimation benchmark for degradation and that the specifics of in vivo degradation phenomena may differ. Nevertheless, this system showed good agreement with parameters seen during in vivo animal studies, including loss of PGA integrity at 2–3 months, and no fibers by histology at 6 months post-implantation^1^.

### Scaffold mechanical characterization

Mechanical testing of ring samples in vitro demonstrated that the TEVG is 433% stiffer than the native IVC at the time of implant as inferred from the slope of an equivalent pressure–diameter curve at physiologic pressures of 5 and 50 mmHg (Fig. 1m). However, the stiffness of the scaffold drastically decreased over the first few months of simulated implantation, consistent with an order of magnitude loss in burst pressure (Fig. 1k). This finding was also consistent with SEM imaging that demonstrated a drastic increase in broken PGA fibers correlating to the loss of stiffness of the TEVG. These data suggest that the rapid loss of the PGA knit architecture leads to a similar rapid loss of scaffold strength. As such, the TEVG initially bears the full mechanical load, stress shielding the cells and neotissue early after implantation. However, this load will quickly be transferred to the neotissue as the PGA fibers degrade and the compliance of the scaffold increases during the first few months following implantation.

### Patient cohort and data collection

The Food and Drug Administration-approved clinical trial (IDE 14127) was designed to evaluate the safety of TEVGs utilized in the extracardiac modified Fontan procedure for single ventricle palliation at which time the IVC is connected to the pulmonary arteries. Patient demographics, the clinical trial protocol, and the associated imaging timeline have been published^20^. Briefly, four patients received TEVGs. Imaging studies included echocardiograms performed monthly during the first postoperative year, at 4-month intervals during the second postoperative year, and annually thereafter. Magnetic resonance images (MRIs) were used to evaluate anatomy and flow at 6 months, 3 years, and 5 years post-implantation. Three of the four patients (Patients 2–4) underwent catheterization and balloon angioplasty of the grafts for stenosis, defined as a reduction in diameter of 50% compared to the nominal diameter at implant^20^. For this cohort, the reduction of minimum graft diameter at intervention time points ranged from 58 to 77% (which corresponded to a cross-sectional area stenosis of 79–94% compared to graft at implant) (Table 1).

**Table 1:** Description of clinical data collected from patients.

	Sex	Age at operation (years)	Graft diameter at implant (mm)	Diagnosis			
Patient 1	F	3	18	Heterotaxy syndrome, unbalanced AVSD, pulmonary atresia			
	Clinical event	Time post Fontan operation (months)	Minimum diameter (mm)	Diameter stenosis (% from implant)	Area stenosis (% from implant)	BSA (m^2^)	Elastic modulus (kPa)
	MRI I	6	18	0%	0%	0.64	13.14
	MRI II	36	9.4	48%	61%	0.74	10.51
	MRI II	60	8.8	51%	63%		
Patient 2	M	2	16	PA-IVS, tricuspid stenosis, HRHS			
	Clinical event	Time post Fontan operation (months)	Minimum diameter (mm)	Diameter stenosis (% from implant)	Area stenosis (% from implant)	BSA (m^2^)	Elastic modulus (kPa)
	MRI I	6	6.8	58%	72%	0.53	16.00
	Catheter lab (cutting balloon angioplasty, LPA stent placement)	7	5.6	65%	84%		
	MRI II	36	7.9	51%	72%	0.67	8.00
	MRI III	60	11.5	28%	36%		
Patient 3	F	3	16	HLHS, mitral atresia/aortic atresia variant			
	Clinical event	Time post Fontan operation (months)	Minimum diameter (mm)	Diameter stenosis (% from implant)	Area stenosis (% from implant)	BSA (m^2^)	Elastic modulus (kPa)
	Catheter lab (cutting balloon angioplasty, LPA stent dilation)	5	6.5	59%	82%		
	MRI I	6	7.1	56%	79%	0.53	16.00
	MRI II	36	7.5	53%	68%	0.67	17.00
	MRI III	60	10.5	34%	71%		
Patient 4	F	4	16	HLHS, mitral atresia/aortic atresia variant			
	Clinical event	Time post Fontan operation (months)	Minimum diameter (mm)	Diameter stenosis (% from implant)	Area stenosis (% from implant)	BSA (m^2^)	Elastic modulus (kPa)
	Catheter lab (cutting balloon angioplasty)	5	3.6	78%	94%		
	MRI I	6	6.8	58%	79%	0.68	14.72
	Catheter lab (cutting balloon angioplasty)	7	6.6	59%	80%		
	MRI II	36	8.6	46%	65%	0.83	13.67

Elastic moduli were tuned to match fraction area change deformation observed at each MRI imaging session.

*AVSD* atrioventricular septal defect, *PA-IVS* pulmonary atresia with intact ventricular septum, *HRHS* hypoplastic right heart syndrome, *HLHS* hypoplastic left heart syndrome.

### Change in TEVG geometry and stiffness by echocardiography

**Fig. 2:**
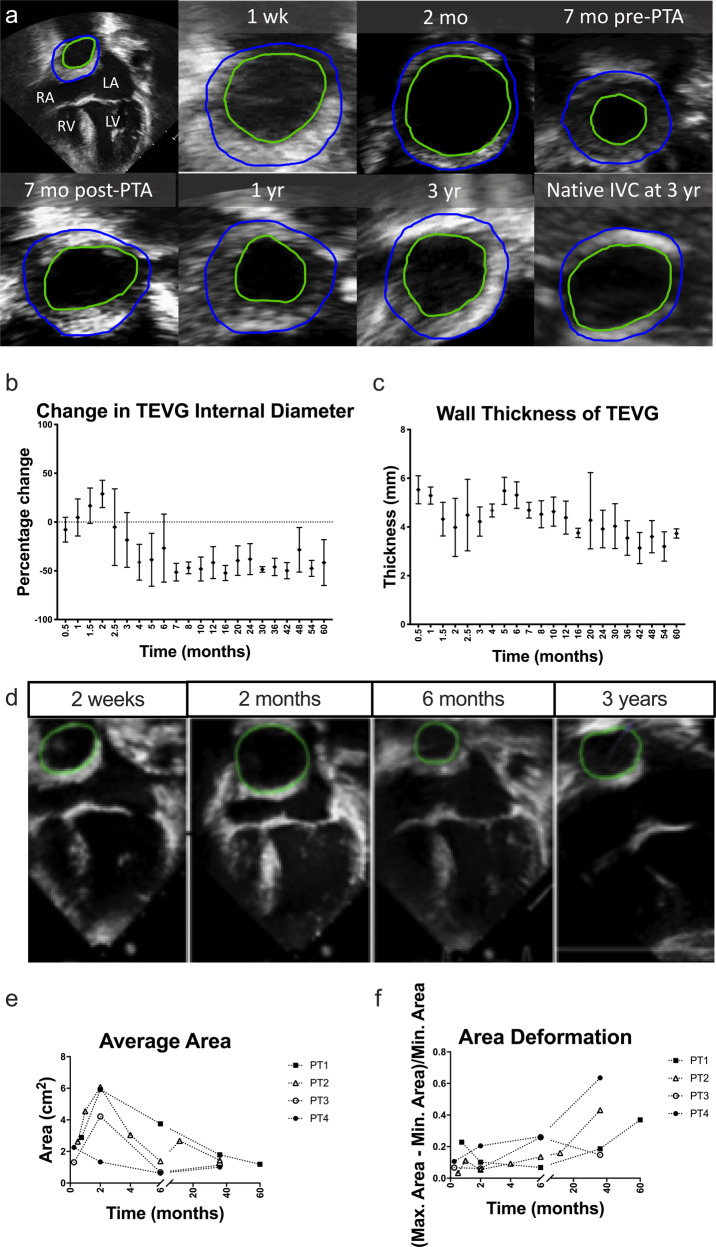
Natural history of neovessel formation in clinical trial: a serial echo analysis of morphometry and biomechanics. **a** The internal and external circumference of the TEVG is labeled and captured in cross-section located lateral and posterior to the atrium for Patient 2 in the U.S. clinical trial. **b** Percent change in TEVG diameter compared to the size at implant. (*n* = 4), error bars represent standard deviation. **c** Changes in TEVG thickness. (*n* = 4), error bars represent standard deviation. **d** Example images of serial changes in TEVG area for PT2 from the clinical trial. **e** Absolute change in area. **f** Graft stiffness over time for all four patients in the clinical trial. PTA percutaneous angioplasty.

Serial changes in graft diameter were calculated from transthoracic echo images performed at routine intervals (Fig. 2). On average, TEVGs were dilated approximately 2 months post-implantation followed by narrowing at 7 months (Fig. 2b). Overall, graft thickness peaked early after implantation followed by a second peak associated with the time of maximal graft narrowing (Fig. 2c). Speckle tracking (TomTec Arena) of the luminal surface allowed quantification of changes in TEVG cross-sectional area across one cardiac cycle. Three out of four patients demonstrated decreased stiffness over time (Fig. 2f).

### Evaluation of TEVG growth and change in graft stiffness by MRI

**Fig. 3:**
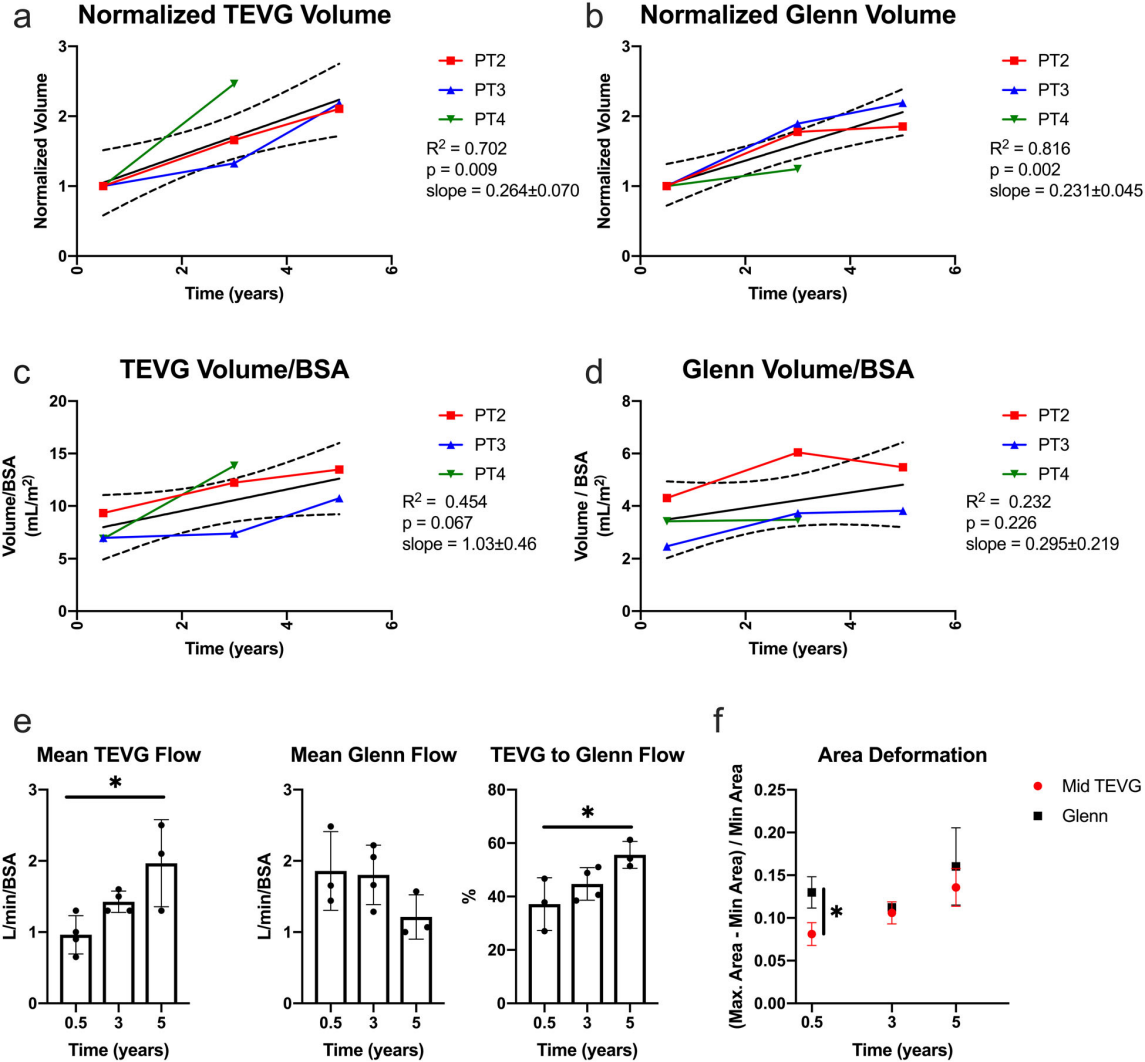
TEVG growth analysis: serial MRI data from the clinical trial. **a** TEVG volume normalized to the size of the graft at 6 months. **b** Glenn volume normalized to the size of the vessel at 6 months. **c** Change in TEVG volume over time normalized to body surface area. **d** Change in Glenn volume over time normalized to body surface area. **e** Mean volumetric flow through the TEVG and Glenn normalized to body surface area. Error bars represent standard deviation. **f** Change in luminal cross-sectional over one cardiac cycle evaluated longitudinally by MRI. (*n* = 4 at 0.5 and 3 year time points, *n* = 3 at 5 year time point) Error bars represent standard deviation. TEVG tissue-engineered vascular graft, Glenn superior vena cava to pulmonary artery connection, BSA body surface area.

The trend seen in the echo data of decreased stiffness (Fig. 2f) was confirmed by MRI (Fig. 3f) with a statistically significant difference in stiffness of the TEVG compared to the native SVC at 6 months. Patient 1 was unique because she had heterotaxy syndrome and possessed a midline IVC, which resulted in a markedly curved (C-shaped) Fontan pathway. In order to prevent graft kinking and avoid external compression, the TEVG was implanted in two pieces anastomosed at an angle. This resulted in the graft being significantly larger than its nominal diameter at the site of the beveled anastomosis. Given the conduit construction unique to this patient and its potential impact on the time course for graft remodeling, an analysis of growth was undertaken excluding this patient. Here we see a statistically significant increase in TEVG and Glenn (SVC to pulmonary artery connection) volumes over time (Fig. 3a, b). Measurements of volume were normalized to the first MRI imaging time point to compare growth over time. The increase in TEVG volume was also compared to that of the Glenn, which served as an internal control for growth. Both the TEVG and Glenn increased in volume over the observed timeframe. An analysis of the volumetric flow through the Glenn and Fontan showed a statistically significant difference between these two structures (Fig. 3e). Evaluation of increases in length normalized to the 6-month MRI time point confirmed long-term increases in graft length across all patients (Supplementary Fig. 2d). Notably, TEVG length was not significantly affected by angioplasty (Supplementary Fig. 2a). Analysis of changes in graft volume including all patients is shown in Supplementary Fig. 2e. Here we can see the large difference in graft volume between Patient 1 and the remainder of the cohort at the first imaging time point with decreases in graft volume out to 3 years from the time of implantation. A comparison of the average TEVG cross-sectional area to surrounding native structures including the SVC, pulmonary artery, and abdominal IVC is shown in Supplementary Fig. 3.

### Patient-specific modeling of TEVG behavior

**Fig. 4:**
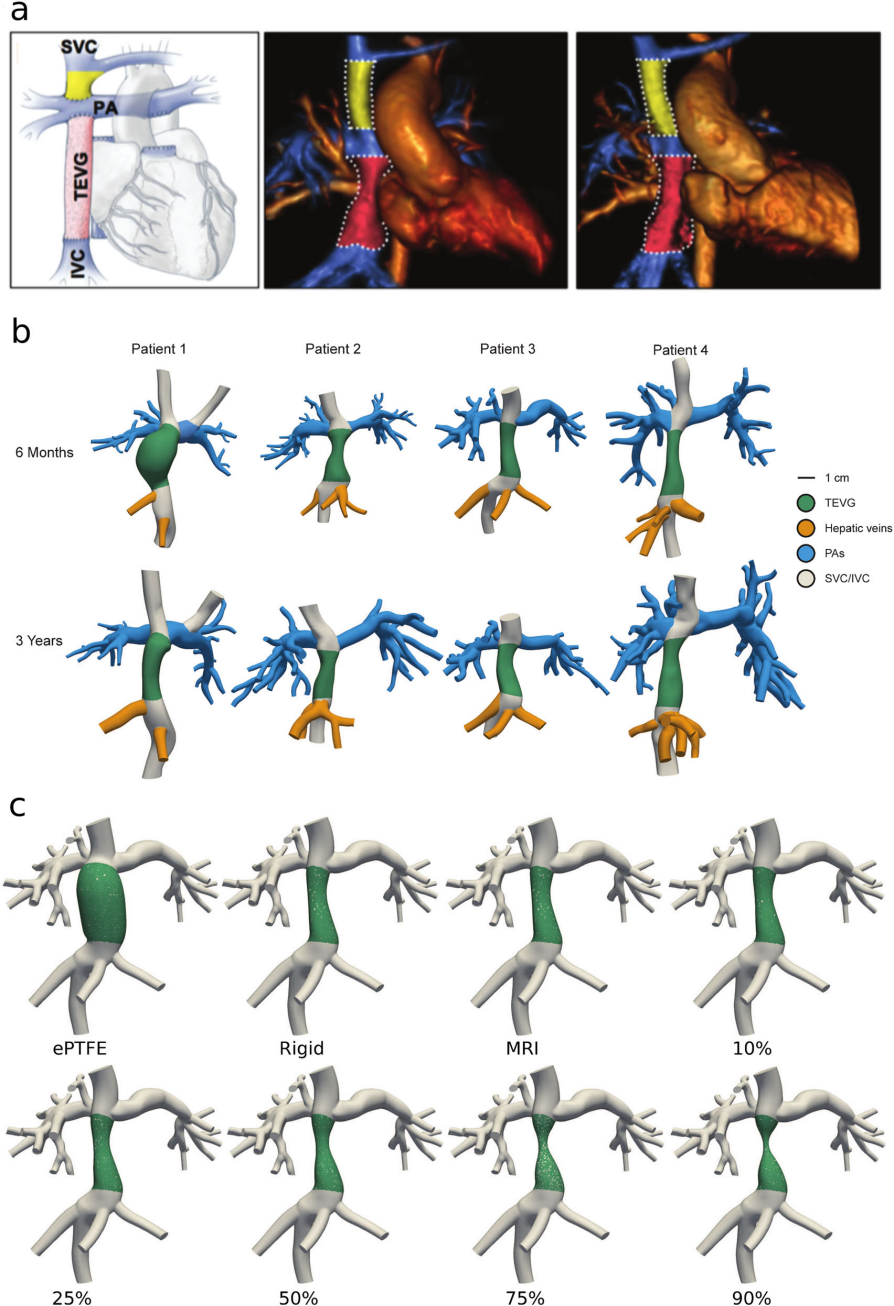
Patient data and CFD model generation. **a** MRI of TEVG and surrounding anatomy at 6 months and 3 years post-Fontan for Patient 2 in the clinical trial. **b** Models of TEVG Patients 1–4 from MRI imaging at 6 months and 3 years post-Fontan time points. **c** Representative geometries of Patient 3 with an artificial ePTFE graft geometry, an assumed rigid graft having the in vivo geometry, and finally the MRI measured TEVG with native geometry and stiffness but simulated degrees of stenosis from 10 to 90% (relative to the measured MRI geometry, not nominal).

From MRI imaging data, patient-specific CFD models were created for all four patients at 6 months and 3 years post-Fontan operation (Fig. 4a, b). The models were modified in silico to mimic varying degrees of stenosis from the time at imaging, with the stenosis placed at areas of observed narrowing or, if there was none, at mid-TEVG. Stiffness of the TEVG was tuned to match observed fractional area change mid-TEVG and ranged from 8 to 14.7 kPa. To compare against the current standard of care, ePTFE grafts with no growth potential, we investigated both the effects of changing the TEVG stiffness to match that of a ePTFE graft and virtually implanting a 16-mm diameter ePTFE graft (Fig. 4c). Patient-specific simulations were run at a youth metabolic equivalent (MET) of 1, 3, and 5 to simulate oxygen consumption states of rest, moderate exercise, and maximum exercise, respectively, using a closed-loop lumped parameter network (LPN) to define inlet and outlet boundary conditions for the 3D hemodynamics within the graft. MET is the ratio of metabolic rate (described as VO_2_) used during an activity to the basal metabolic rate (6.5 ml/kg/min in children)^21^. MET 5 is a standard maximum metabolic rate found in non-TEVG Fontan children. Details of the simulation parameters are described in the “Methods” section. Oxygenation metrics, systemic/venous metrics, and local hemodynamic metrics describing the in silico performance of each model were then calculated from the simulation data. For this study, the MRI datasets are used to model patient geometry and the time points are considered independent to investigate the effects of geometric changes.

Data were plotted by fitting a two-term exponential on top of the simulated dataset for visualization purposes. Wilcoxon signed-rank tests showed significant differences in calculated metrics at all stenosis levels compared to the geometry at MRI.

### Validation of TEVG simulations

**Fig. 5:**
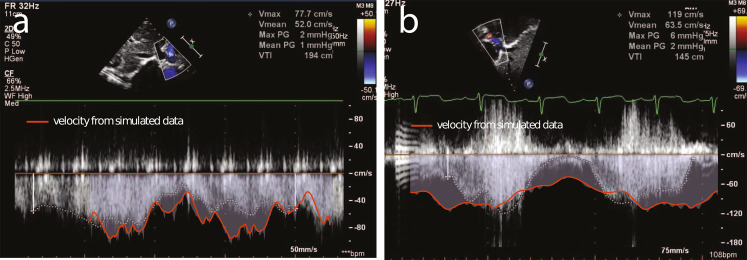
Validation of simulated stenosis against echocardiograph data of in vivo stenosed grafts. **a** Patient 2 at 6 months post-implantation (graft diameter of 5.6 mm by echocardiogram) measured echo velocity compared to Patient 2 at 6 months (simulated stenosis of 6.0 mm graft diameter) modeled velocity. **b** Patient 3 at 5 months post-implantation (graft diameter of 6.5 mm by echocardiogram) measured echo velocity compared to Patient 3 at 6 months post-implantation (simulated stenosis of 6.2 mm graft diameter) modeled velocity.

To validate our LPN’s ability to recreate the performance of stenosed geometry, we compared the velocities predicted by our simulations with the velocities measured via echocardiography. Each echocardiogram was paired with the simulation that closely matched its post-implantation time point and estimated stenosis level. The resulting comparison to the echocardiograms indicated good agreement between clinically measured velocity and our simulations (Fig. 5). Additional comparisons of simulation velocity to clinically measured echocardiagram velocity are provided in Supplementary Fig. 4.

### Oxygenation metrics

**Fig. 6:**
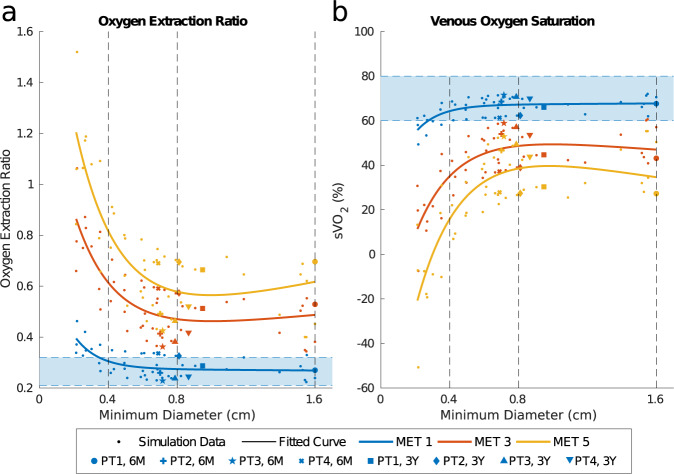
Oxygenation metrics describing the performance of simulated graft geometries. **a** Estimated oxygen extraction ratio (OER) at MET levels with MET 1 reference values of 0.21–0.32 OER indicated by the shaded region. **b** Estimated sVO_2_ at MET levels with MET 1 reference values of 60–80% sVO_2_ indicated by the shaded region.

To compare the adequacy of oxygen delivery that would result from each model, the oxygen extraction ratio (OER) was estimated from the simulations (Fig. 6a). At rest, for minimum graft diameters >4 mm, OER remained within the normal range (0.21–0.32, marked in Fig. 6a)^22^. At both MET 3 and MET 5, OER increased above this range, consistent with behavior during exercise^23^. ePTFE graft geometry, 16 mm in Fig. 6, did not have a large impact on OER and produced similar values as the TEVG. This suggests that, even with a 90% reduction from the luminal cross-sectional area at implant, TEVGs are comparable to ePTFEs for OER across MET levels.

To determine the effect of each model on overall blood oxygen levels, mixed venous oxygen saturation (sVO_2_) was also estimated from the simulations. sVO_2_ values for all patients at MET 1 stayed within the expected range of 60–80% up to a 4-mm minimum diameter (Fig. 6b)^22^. At MET 3 and MET 5, stenosis had a much higher impact on sVO_2_, with the perturbation from the TEVG reference geometry increasing with MET level. These findings suggest that the degree of graft narrowing experienced in the clinical trial could be well tolerated, consistent with the lack of significant clinical symptoms. Notably, at MET 5 and minimum diameter <3 mm, sVO_2_ reaches a negative value implying that this level of MET is not physiological. sVO_2_ in the ePTFE graft geometry were slightly lower than in the TEVG reference geometry at MET 3 and MET 5, although the difference was small relative to the overall value (<0.7%).

### Systemic/venous metrics

**Fig. 7:**
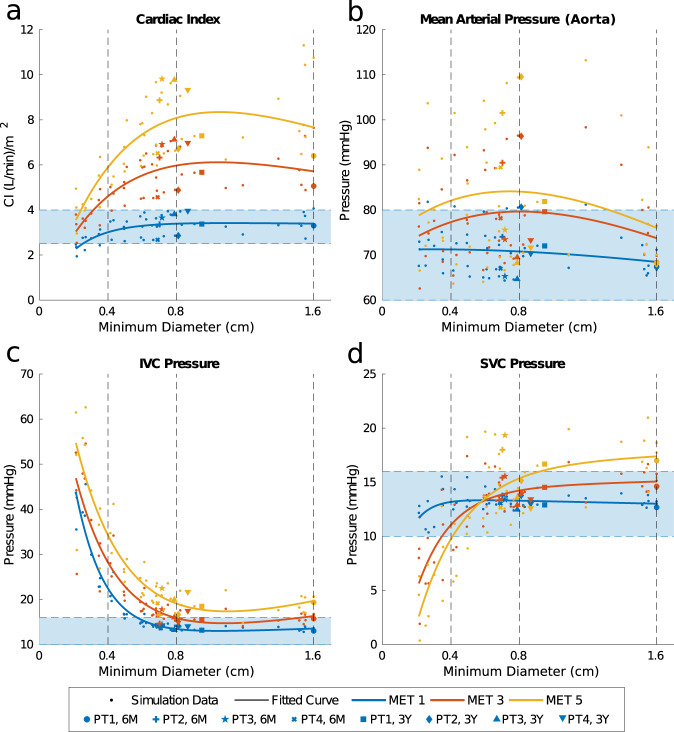
Systemic and venous metrics describing the performance of simulated and MRI measured graft geometries in terms of cardiac index (CI), arterial pressures, and venous pressures. **a** Estimated CI at MET levels with a MET 1 reference values of 2.5–4.0 L/min/m^2^ indicated by the shaded region. **b** IVC pressures at MET levels with a MET 1 reference values of 10–16 mmHg indicated by the shaded region. **c** MAPs at MET levels with a MET 1 reference values of 60–80 mmHg indicated by the shaded region. **d** SVC pressures at MET levels with a MET 1 reference values of 10–16 mmHg indicated by the shaded region.

Cardiac index (CI), an important measure of ventricular function as well as venous return, was affected by stenosis level at both rest and exercise. Greater stenosis caused decreasing CI values, with the percentage decrease from nominal geometry proportional to MET level (Fig. 7a). However, this did not cause the predicted CI to decrease below the threshold for normal CI values until a minimum graft diameter <4 mm^22^. For the simulated ePTFE grafts, CI was only slightly higher than TEVG MRI geometry at rest, but it was notably larger than the reference models at MET 3 and MET 5. The mean increase in CI at these levels was 0.21 and 0.43 L/min/m^2^, respectively.

Arterial and venous pressures, for which long-term deviation from normal values can lead to clinical sequelae including organ damage, were also mediated by stenosis although only in certain areas within the body (Fig. 7). Mean systemic arterial pressures (MAPs) measured in the aorta and SVC pressures remained within normal ranges for Fontan patients at minimum diameters as low as 3 mm, whereas IVC pressures increased above normal values at 5 mm minimum diameter and below^24,25^. In general, pressure values proximal to the stenosis were impacted more than those distal and those in the upper body. MET level did not intensify the impact of stenosis on pressures in terms of their percent increase or decrease from MET 1 values.

### Local hemodynamics

**Fig. 8:**
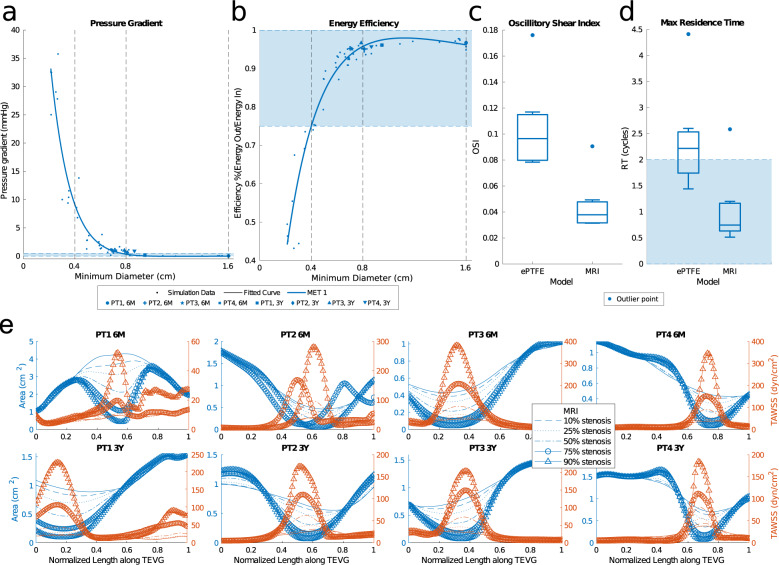
Local hemodynamics of simulated and MRI measured graft geometries. **a** Pressure gradient across the Fontan graft at MET1. **b** Energy efficiency of the TCPC at MET 1 with reference values of 75–100% indicated by the shaded region. **c** Box plot of OSI at MET 1 showing quartiles and outliers. **d** Box plot of max RT at MET 1 showing quartiles and outliers. **e** Wall shear stress compared to cross-sectional area over the length of TEVGs at various stenosis levels.

Pressure gradients were quantified across the models at MET 1 (Fig. 8a). Pressure gradients in the Fontan graft are used as a metric of graft performance, and gradients as low as 0.5 mmHg can be a clinical indicator for intervention. In our simulations, the median gradient increased exponentially with stenosis level. However, the existence of pressure gradients did not necessarily indicate a decrease in hemodynamic performance. Many models with up to a 4 mmHg pressure gradient performed well within the expected values for calculated systemic/venous and oxygenation metrics (Figs. 6 and 7).

Energy efficiency of the total cavopulmonary connection (TCPC) decreased with increasing stenosis at MET 1 but was only below normal values of extracardiac TCPC energy efficiency at <4 mm minimum graft diameter (Fig. 8b)^26^. ePTFEs (16 mm diameter) had a higher energy efficiency than the TEVG reference geometry, although the mean magnitude of this difference was small compared to the overall energy lost in the models (<5%).

Abnormal values of wall shear stress can trigger maladaptive vascular responses and influence growth and remodeling. When averaged across the graft surface, there was no significant difference in the time-averaged wall shear stress (TAWSS) across stenosis levels, except in the ePTFE graft models that had significantly lower TAWSS values, which can trigger platelet activation and changes in endothelial cell biology^27^. There is a stronger relationship between stenosis and TAWSS in the area immediately surrounding a stenosis, where TAWSS monotonically increased with increased stenosis (Fig. 8e).

To determine the risk of thrombosis and quantify the presence of blood recirculation inside the graft, we computed maximum residence time (RT), the maximum time a particle remains in the volume of interest on average, and the oscillatory shear index (OSI). We compared these values for the ePTFE grafts (16 mm diameter) and the actual TEVG geometries observed at the 6-month and 3-year MRI time points (6.9–16 mm minimum diameter). Max RT and OSI were three times larger in ePTFE graft models than in TEVG reference geometries (Fig. 8c, d). Visualizing the streamlines show that the primary mechanism responsible for the increased RT is flow separation at the IVC anastomosis that leads to large curvature against the dilated graft wall and small pockets of recirculation.

Local hemodynamics, including a representative streamline trace, are visualized for a sample patient in Supplementary Fig. 6.

## Discussion

Traditional biomaterials for clinical use in surgical treatments of congenital heart diseases are limited by their lack of growth capacity and durability^2^. Our TEVGs are constructed from a biodegradable polymeric scaffolds that are seeded with bone marrow-derived mononuclear cells^6^. Replacement neotissue forms in vivo through the host immune response as the scaffold degrades. We have experimentally demonstrated, in both small and large animal models, two distinct phases of neotissue formation: an early inflammation-driven response, which is dominated by the foreign body response to the polymer, followed by mechano-mediated tissue remodeling as the scaffold degrades, lessening the foreign body response and exposing the resident cells to increasing mechanical load^20^. First-in-human clinical evaluation of this graft technology was undertaken in Japan, which confirmed the growth capacity of the conduit and identified stenosis as the primary graft-related complication. Application of this same graft technology in a U.S. clinical trial, with increased frequency of imaging within the first year of implantation, found an unexpectedly high rate of stenosis^20^. Although most of the patients were clinically asymptomatic, three of the four patients received balloon angioplasty to relieve the graft stenosis based on a >50% reduction of cross-sectional area from the graft at implant^20^. Subsequent preclinical large animal testing motivated by computational models demonstrated that, while stenosis is common, it typically is well tolerated and reverses spontaneously in most cases^20^. Despite the limitations inherent in these animal models, as no viable models recapitulate single ventricle physiology, this finding questioned whether we were overly aggressive in the treatment of our patients in the U.S. clinical trial and defined a pressing need to further evaluate the effects of graft stenosis particularly on the hemodynamics that are central to procedural success or failure.

To better capture the dynamic changes in graft morphology and its effect on graft function, we analyzed TEVG behavior and performance both soon and well after implantation. Key observations include graft dilation at 2 months (Fig. 2b), which corresponded approximately to the estimated loss in mechanical integrity of the polymeric scaffold (Fig. 1i, k, n). Subsequent graft narrowing is generally noted at 6–7 months post-implantation (Fig. 2b) which was due to wall thickening resulting in luminal narrowing. Previous computational–experimental work by our team suggest that this is due to an inflammation-driven mechano-mediated process^20^. Regardless of such mechanisms, following angioplasty there no recurrence of stenosis beyond 1 year. In evaluating graft distensibility, we note the graft transitions from a stiff polymeric scaffold to a neovessel having structural properties similar to the native vein (Figs. 2f and 3f). The decrease graft stiffness and presumed improved compliance is important to note when evaluating graft size. The IVC is a capacitance vessel that can increase in size based on the preload and hemodynamic state. To exclude the confounding variable of compliance on measurement of vessel size, we evaluated changes in TEVG length. Importantly, we noted a statistically significant increase in length in all patients (Supplementary Fig. 2d). Results of the volumetric growth analysis were found to be statistically significant with the exclusion of Patient 1, who had a unique graft construction secondary to distinct anatomic considerations and whose graft demonstrated a variable time course for remodeling in comparison to the remainder of the cohort. Whether the difference seen in the time course of remodeling relates to the construction of the graft or in variation of the remodeling response across patients is difficult to determine given the small sample size. We normalized the growth of the grafts to the 6-month MRI time point as preclinical small and large animal work demonstrated complete scaffold degradation by approximately 6 months^20,28–31^. We submit that it is not reasonable to expect any degree of long-term growth while the polymeric scaffold retains mechanical integrity, but how much and at what rate should we expect to see significant growth? This is a challenging question to directly determine as in the normal vascular anatomy there is no native structure that connects the IVC to the pulmonary arteries. The Glenn likely serves as the best internal control of growth of a native vascular structure as it is similar to the TEVG in having been anastomosed to the pulmonary arteries and has been surgically manipulated. However, the proportion of growth of the upper and lower body (and thus the amount of cardiac output draining to the SVC and IVC) changes over time and may exhibit differential growth. Evaluation of the split in volumetric flow between the upper and lower body in our patient cohort is consistent with the expected trend of the lower body increasing in size over time to a greater extent than the upper body (Fig. 3e). Comparing the growth of the TEVG and Glenn normalized to body surface area demonstrated a trend of larger increases in the TEVG suggesting that growth may be mediated by changes in body size or blood flow through the graft (Fig. 3c, d). Qualitatively, it is striking to note that by 3 years from implantation the graft matches the size of the native IVC to which it is anastomosed (Fig. 4b). This holds true for all patients, independent of the time course for graft remodeling or previous balloon angioplasty.

CFD has successfully been used in clinically relevant evaluations of interventions and treatment planning for congenital heart disease. In Fontan palliation, CFD has played an important role in the transition in surgical methods from the atriopulmonary connection to the TCPC, now the standard of care, by providing evidence for even flow splits and reduced energy losses^32–37^. It has also been used to propose novel TCPC geometries that have been translated to clinical use^38,39^. Here we applied CFD to quantify the effects of changes in TEVG geometry and stiffness on metrics that can inform clinical decisions. As demonstrated in this study, computational modeling provides a means to quickly and efficiently evaluate a range of graft anatomies and quantify effects on hemodynamics and physiology.

Our modeling-based evaluation demonstrates that Fontan physiology is robust to large changes in TEVG geometry, including the majority of the narrowing observed clinically in our patient cohort. At 6 months and 3 years post-Fontan operation, mid-TEVG cross-sectional area was on average 67% less than that at implant (2 cm^2^) and minimum TEVG diameter was on average 56% less than that at implant (16–18 mm). Despite these significant geometric changes, our models at these time points maintained relatively consistent hemodynamic performance metrics, remaining well within the expected physiological range for Fontan patients. Significant impacts to these metrics were not observed until high stenosis levels of approximately 86% cross-sectional area reduction from the TEVG at implant. The high degree of stenosis required to impact patient hemodynamic performance is in agreement with clinical observation; patients in our study were generally asymptomatic and rarely reached the levels of stenosis predicted to significantly change performance. One patient in our study did develop stenosis approaching the critical level observed in our modeling. Patient 4 at 5 months post-implantation developed a stenosis with a 95% reduction in cross-sectional area compared to the size at implantation. This patient reported symptoms of chest pain and fatigue, in agreement with the computational threshold we identified.

While patient-specific simulations hold promise for evaluating stenosis risk, there is a need for quantitative guidelines on how minimum diameter affects Fontan performance. While it is difficult to predict a universal minimum diameter threshold for indicating clinical concern in the context of each patient’s unique anatomy and physiology, an estimate can be made from the trendlines of the patients in this study. Based on the region that most metrics remained within normal Fontan range, a 4-mm minimum diameter appears to represent a reasonable threshold based on our findings and clinical observations. The existence of a pressure gradient in the Fontan graft is also a quantitative measurement used clinically to evaluate graft function. Even gradients as low as 0.5 mmHg are sometimes considered a cause for concern, but our simulations suggest that grafts with up to 4 mmHg pressure gradients perform well within normal ranges for metrics such as OER, sVO_2_, systemic/venous pressures, and local hemodynamics. Importantly, however, our model tended to overestimate the values of pressure drops of stenosed models when compared to the measured value at catheterization^20^. While for Patient 2, Patient 3, and the second catheterization of Patient 4, the model predicted the pressure drop within 1 mmHg; for the first catheterization of Patient 4, our model predicted an approximately 10 mmHg pressure drop vs the 3.5 mmHg pressure drop observed clinically. This may be due to a variety of factors, including (i) anesthesia during the catheterization procedure decreasing MET value <1 and (ii) a lack of modeling physiologic compensatory mechanisms in response to graft stenosis beyond increased blood volume. This would include phenomena such as collateral vessel formation (which Patient 4 had at first catheterization). While this suggests a limited ability to predict high pressure gradients, it also suggests that our model may present upper bounds on observable pressure gradients due to the lack of additional stenosis-regulatory mechanisms. Liver pressure is also sensitive to changes in minimum graft cross-sectional area, and while it is generally within the normal range for Fontan patients with a minimum graft diameter >5 mm, individual patients may see elevation in liver pressure and be at risk for liver fibrosis, a known complication associated with Fontan physiology and worsened by elevated venous pressure. Thus, even small increases in the liver pressure may be of clinical significance and warrant close observation independent of the global circulatory hemodynamics at rest and exercise^40^.

Our findings also reveal limits to the physiological mechanisms that compensate for increased TEVG resistance. Below a 4-mm minimum diameter, observations of OER values >100% and sVO_2_ values <0% at MET 5 indicate that these patients would not realistically reach this level of exercise at high stenosis levels. This is primarily due to the inability to increase cardiac output despite the imposed MET and subsequent changes to circulatory parameters. As MET 5 is a typical maximal exercise rate, being unable to reach this value in silico predicts reduced maximum exercise capacity.

Simulations of virtually created stenosis geometries were validated by comparisons to echocardiogram velocity. There was good qualitative agreement between the echocardiogram and simulated velocity waveform (which represented the maximal velocities recorded in silico). Within the general agreement between modalities, the simulations tended to predict higher values than the echocardiograms. This is expected as the magnitude of the measured velocity is reduced if the angle of assessment of the echocardiogram is not well aligned with the velocity direction and manual tracing of echocardiograms ignore sporadic peaks. Considering this, as well as the confidence interval on echocardiogram velocities and the fact that we cannot replicate the exact positioning of the ultrasound probe, the simulated values match the clinically observed values well. The stiffness of the grafts in silico were tuned to match deformation observed in the phase contrast (PC) deformation after applying flow and pressure amplitude boundary conditions from PC-MRI and catheter data, respectively. The resulting range of stiffness values closely matched the stiffness values observed in the degradation study, supplying additional validation for this method of wall property tuning (Fig. 1l and Table 1).

TEVGs share similarities with traditional ePTFE grafts in both geometry and deformation, especially when they are first implanted. However, as the TEVGs evolve into neovessels, differences become more dramatic and it is important to understand how TEVGs differ in performance from the standard of care. Just changing the stiffness of the TEVG in simulations to be rigid, mimicking ePTFE while maintaining the same shape as the TEVG, had no significant effect on the global or local hemodynamics (Table 2). Widening the conduit diameter to 16 mm and assuming a rigid wall had no effect on the global flow at MET 1 and had minimal impact on simulation results at MET 3 and MET 5. These differences were seen in CI, OER, and sVO_2_, although the overall magnitude of these differences was not large (Figs. 7a and 6 and Table 2). The small magnitude of these differences supports the idea that narrowing of the TEVG does not impede oxygen delivery or overall circulatory physiology even at higher levels of stenosis. However, the local hemodynamics of the wider ePTFE graft were significantly different. We observed significantly higher energy efficiency and lower pressure gradients. Like CI, OER, and sVO_2_, these differences from TEVG reference geometry models were significant but were not large in magnitude. The most striking differences in the ePTFE graft were in OSI and RT. The threefold increase in max RT observed in the ePTFE graft models and visualization of streamlines implies the presence of flow separation and recirculation in the oversized graft that is not present in the TEVGs at 6 months and 3 years post-implantation. The higher RT and OSI values also suggest increased thrombotic risk in ePTFE grafts. In particular, average RTs above two cardiac cycles in the coronary/systemic circulation have been linked to higher risks of thrombosis, which regions in most of our ePTFE grafts exceed^41^. Thromboembolic events are a long-term complication following the Fontan operation with the current standard of care^11^. Their cause is likely multifactorial given the associated liver disease and attendant disturbances in coagulation factors reported in Fontan patients. Additionally, the varied application of anticoagulation and antiplatelet therapy makes interpretation of results challenging. More so, the ePTFE material may predispose the graft to clot formation as complete endothelialization of the graft at its midportion may not be achieved given physiologic limitations of human endothelial cell migration^42–44^. In the Japanese clinical trial, a histologic evaluation of our TEVG graft in a patient who died of non-graft related complications 12 years post-graft implantation demonstrated endothelialization along the entire length of the graft^45^. While at early time points thromboembolic risk in the TEVG may be similar to that of an ePTFE due to the short-term dilation and limited cell infiltration, we postulate a lower long-term reduced risk of thromboembolic complications given endotheliazation of the TEVG and decreased RT from a geometry that more closely approximates that of the native vasculature. This would be in agreement with our previous lamb studies where there was no evidence of thrombosis in our TEVGs across many implants^20^.

**Table 2:** Physiologically relevant metrics derived from CFD simulations; results are summarized for a ePTFE (16 mm diameter), rigid wall TEVG, and TEVG geometry from MRI at 6 months and 3 years post-Fontan operation (MRI).

	ePTFE	Rigid	MRI
Minimum diameter (cm, mean ± SD)	1.49 ± 0.14	0.96 ± 0.50	0.96 ± 0.50
OER MET 1 (mean ± SD)	0.27 ± 0.04	0.27 ± 0.04	0.27 ± 0.04
OER MET 3 (mean ± SD)	0.45 ± 0.09	0.47 ± 0.09	0.47 ± 0.09
OER MET 5 (mean ± SD)	0.54 ± 0.12	0.58 ± 0.12	0.58 ± 0.12
sVO2 MET 1 (%, mean ± SD)	67 ± 4	67 ± 4	67 ± 4
sVO2 MET 3 (%, mean ± SD)	50 ± 9	48 ± 9	48 ± 9
sVO2 MET 5 (%, mean ± SD)	41 ± 12	38 ± 11	38 ± 11
CI (L/min/m^2^, mean ± SD) MET 1	3.42 ± 0.47	3.38 ± 0.44	3.37 ± 0.44
CI (L/min/m^2^, mean ± SD) MET 3	6.22 ± 1.21	5.94 ± 1.03	5.94 ± 1.03
CI (L/min/m^2^, mean ± SD) MET 5	8.72 ± 1.90	8.09 ± 1.50	8.09 ± 1.50
IVC MET 1 (mmHg, mean ± SD)	13.49 ± 0.41	13.71 ± 0.52	13.71 ± 0.50
IVC MET 3 (mmHg, mean ± SD)	15.48 ± 0.92	16.09 ± 1.11	16.09 ± 1.11
IVC MET 5 (mmHg, mean ± SD)	18.12 ± 1.93	19.27 ± 2.13	19.27 ± 2.13
SVC MET 1 (mmHg, mean ± SD)	13.28 ± 0.46	13.12 ± 0.48	13.14 ± 0.48
SVC MET 3 (mmHg, mean ± SD)	14.75 ± 1.10	14.23 ± 0.97	14.23 ± 0.97
SVC MET 5 (mmHg, mean ± SD)	16.60 ± 2.62	15.62 ± 2.53	15.62 ± 2.53
MAP MET 1 (mmHg, mean ± SD)	70.60 ± 5.25	70.18 ± 5.34	70.25 ± 5.37
MAP MET 3 (mmHg, mean ± SD)	80.05 ± 9.88	78.66 ± 10.01	78.66 ± 10.01
MAP MET 5 (mmHg, mean ± SD)	85.45 ± 15.76	83.27 ± 15.62	83.27 ± 15.62
Energy efficiency (%, mean ± SD)	97 ± 2	95 ± 1	95 ± 1
Pressure gradient (mmHg, mean ± SD)	0.05 ± 0.05	0.70 ± 0.41	0.71 ± 0.41
Oscillatory shear index (mean ± SD)	0.10 ± 0.03	0.04 ± 0.02	0.05 ± 0.03
Residence time (cycles, mean ± SD)	2.36 ± 0.93	—	1.02 ± 0.68

*SD* standard deviation.

The simulation results in this study point to the mechanism by which stenosis may spontaneously resolve in the months following the observed minimum diameter. Murine models have suggested that stenosis formation in TEVGs is driven by an early inflammatory response during the immediate post-implantation period, which lessens as the scaffold degrades^46^. As shown in Fig. 8e, TAWSS levels increase locally with increased stenosis. The first invariant of the Green strain (indicating TEVG deformation over a cardiac cycle) followed a similar trend, where the graft-averaged value remained constant across stenosis levels, but increased proximal to the stenosis at high stenosis levels (Supplementary Fig. 5). In the presence of an intact endothelium, increased WSS mediates vessel diameter via increased production of endothelial-derived nitric oxide. This reduces synthesis of extracellular matrix, which in turn could attenuate continued thickening and narrowing^46–48^. The increased WSS caused by geometric changes associated with inflammation-mediated stenosis may then drive a subsequent increase in diameter after the influence of the foreign body response fades. This reversal would be expected to occur within the months after implantation, in agreement with the trends in geometric changes seen here. The role of WSS in TEVG evolution should be explored further in future studies that integrate a comprehensive growth and remodeling formulation with fluid–structure interactions. Such a fluid–solid–growth framework could then predict how local hemodynamics shape TEVG geometry and properties over time, thus providing further insight into the mechanism of the observed dilation, stenosis, and eventual equilibrium. The resulting patient-specific predictive framework for growth and remodeling could then be coupled with the in silico framework outlined in this paper to personalize future TEVG graft designs at reduced risk to the patient.

It is important to note that this study represents findings based on a small sample size. While previous clinical application of this technology has confirmed long-term growth of the graft in length, future study is warranted to further confirm the long-term growth potential and the possibility of reversible graft stenosis. In addition, simulations of stenosis geometries only take into account the regulatory mechanism of increased blood volume to regularize aortic pressure and do not model other physiological changes associated with stenosis such as increased heart rate or the development of collateral blood flow. While the validation of our results against the echocardiogram indicates that our simulations reflect accurate physiological waveforms even at simulated geometries, measuring velocity via echocardiogram has limited accuracy. In future studies, simulations could be compared against velocity waveforms from higher resolution modalities, such as PC-MRI.

In this study, we have demonstrated several possible advantages of the use of TEVGs as Fontan conduits, including long-term growth potential and reduced thromboembolic risk. However, these benefits must be weighed against the early risks of graft dilation and narrowing in order to justify their use. We have also quantitatively shown that the primary graft-related complication, stenosis, is well tolerated up to most clinically observed levels. Future use of TEVGs in Fontan patients and other forms of congenital heart disease will further our understanding of how TEVGs can be used to replace traditional biomaterials and advance treatment of congenital heart disease as well as other cardiovascular conditions.

## Methods

### Clinical trial

The clinical trial flow chart and summary have been included in a prior publication^20^. Informed consent was obtained from all human participants. Institutional review board (IRB) approval was granted by Yale University (Human Investigation Committee #0701002198) and Nationwide Children’s Hospital (IRB12-00357 and IRB15-00013). The clinical trial was performed under FDA IDE 14127 in compliance with good clinical practice guidelines.

### TEVG fabrication

The scaffolds (Gunze Ltd.) in the clinical trial were made from PGA fibers and a copolymer of PCLA. Quantitative SEM demonstrated an inner surface average pore size of 41.9 ± 2.7 μm and porosity of 0.87 ± 0.01; the outer surface average pore size was 36.4 ± 6.6 μm and had a porosity of 0.86 ± 0.02^20^. On the day of implantation, bone marrow (5 mL/kg body weight) was harvested from the patients and the mononuclear cell fraction was isolated using density centrifugation^6^. The mononuclear cells were seeded onto the scaffold using a custom vacuum system. The scaffold was incubated in autologous plasma for 2 h prior to implantation on the same day the TEVG was assembled^20^.

### Scaffold degradation

Five-mm-long samples were cut from 16 mm diameter scaffolds (*N* = 3/group) for use in degradation testing. Samples were weighed and then submerged in 20 mL 1× PBS (pH 7.4) at 70 °C for 5 min (0 days), 1 day, 2 days, or 3 days. 70 °C was chosen to accelerate degradation to simulate 1 month in vivo degradation with 1 day in vitro degradation (calibration data not shown). After degradation, samples were washed twice with dH_2_O for 5 min to remove residual salts and then frozen to −80 °C and lyophilized overnight. Dry weights were measured and compared to pre-degradation weights. Five-minute samples were used as 0 day controls for polymer swelling effects of fluid.

### Compliance testing

The initial porosity of the scaffolds precluded standard pressure–diameter testing. Hence, following degradation, ring samples were mounted on a thin rod. A slotted weight set with hanger was hung from the bottom side of the ring sample, and the resulting length, *L*, of the sample was measured. Successive weights were added and lengths were recorded until sample failure. Hanging weight was correlated to pressure as: *P* = *mg*/(2*wr*), where *P* = pressure, *m* = hanging mass, *g* = gravity, *w* = width (along the axial direction) of sample, and *r* = internal radius of the ring sample. Hanging length, *L*, was correlated to radius as *r* = *L*/*π*. Stiffness could be inferred from the stress–stretch response, namely, *mg*/(2*wh*) vs *L*/*L*_o_, where *h* is sample thickness and *L*_o_ is the original length (with thickness determined from incompressibility from original volume as *V*_o_ = 2*πrwh* or *h* = *V*_o_/(2*πrw)*). Burst pressure was defined by the weight that caused sample failure.

### SEM analysis

Degraded ring samples were cut along the axial direction to create 0.5 cm squares for image analysis. In all, 0.5 × 0.5 cm square samples were mounted on SEM stages with carbon tape lumen side up. Samples were sputter coated under vacuum to 3 nm thickness with gold in argon gas. Samples were imaged on a Hitachi S4800 SEM at 5 kV and 10 mA. SEM images were analyzed with the FIJI image analysis software. Fiber diameter was calculated by an average of at least five PGA fibers per image.

### Echocardiography

Patients were followed routinely with transthoracic echocardiograms (Phillips iE33) to evaluate graft size, patency, and fractional area change across a cardiac cycle.

#### Cross-sectional diameter

Echocardiographic images were obtained in the apical four-chamber view and the TEVG was imaged at the level of the right upper pulmonary vein and atrium. Two orthogonal measurements were obtained for each subject. Values were then expressed as a percentage of nominal dimensions from implanted graft size. Each clinical patient had some variability in the postoperative interval for imaging and thus images were grouped into time intervals where at least three patients had images available for quantification. Average values were used for patients with more than one set of images within the time interval in order to not skew results given small numbers.

#### Graft thickness

Images for measurement were obtained from the apical four-chamber imaging plane with the TEVG in cross-section at the level of the insertion of the RUPV into the atrium as a landmark. Measurements were performed in triplicate across the atrial side of the TEVG where wall thickness was able to be easily visualized. Measurements encompassed both a portion of the TEVG as well as the thickness of the atrial wall. Given that the atrial wall is consistently thin, it is logical to conclude that changes in thickness are most likely related to the remodeling of the TEVG. Triplicate measurements were averaged, grouped according to the same time intervals as utilized for the graft dimensions. Data are displayed as mean + SD.

#### Area deformation

An apical four-chamber view of the heart at the level of atrioventricular valve opening was captured to profile the tissue-engineered graft in cross-section. Speckle tracking echocardiography (Image Arena, TomTec Imaging Systems) was utilized to label and track the graft across one cardiac cycle. Post-processing included calculation of absolute area and area deformation (defined as maximum area minus minimum area divided by minimum area)).

### Magnetic resonance imaging

Cardiac MRI was performed with a 1.5- or 3-Tesla magnet utilizing 8- or 32-channel phased-array cardiac coil. Sequences included steady-state free precession (SSFP) 2-chamber, 3-chamber, 4-chamber, and short axis stack. In order to fully examine the tissue-engineered Fontan graft, SSFP gated sequences were done for axial and coronal stacks through the graft. Additional double inversion recovery sequences were performed in the axial, sagittal, and coronal planes. Real-time axial and sagittal cine sequences were performed. Velocity-encoded PC was performed through the ascending aorta, descending aorta, SVC, inferior Fontan, mid-Fontan, superior Fontan, RPA, and left pulmonary artery. Contrast (Gadavist or Ablavar) was injected intravenously for magnetic resonance angiography (MRA). Initially, a 25-phase time-resolved contrast kinetics (TRICKS) sequence was done. This was followed by single-phase MRA sequences in coronal and axial planes. The patient was imaged with free-breathing technique. Conscious sedation was provided by the pediatric anesthesia service. Linear regression of the growth normalized to the first 6-month time point was undertaken with the *P* value corresponding to an *F* test to evaluate for a non-zero slope. MRI volumetric flow data and area deformation are presented as mean with associated standard deviation in Fig. 3e, f. Volumetric flow comparisons were made using analysis of variance with post hoc Tukey’s test for multiple comparisons. *P* values <0.05 were considered significant.

### Model generation

The open-source software package SimVascular (simvascular.org) was used to create 3D anatomic models from the MRI imaging data at two time points for each patient, 6 months and 3 years post-Fontan operation (Fig. 4b)^49^. These models were virtually modified in SimVascular to display varying degrees of stenosis.

### CFD simulations and boundary conditions

**Fig. 9:**
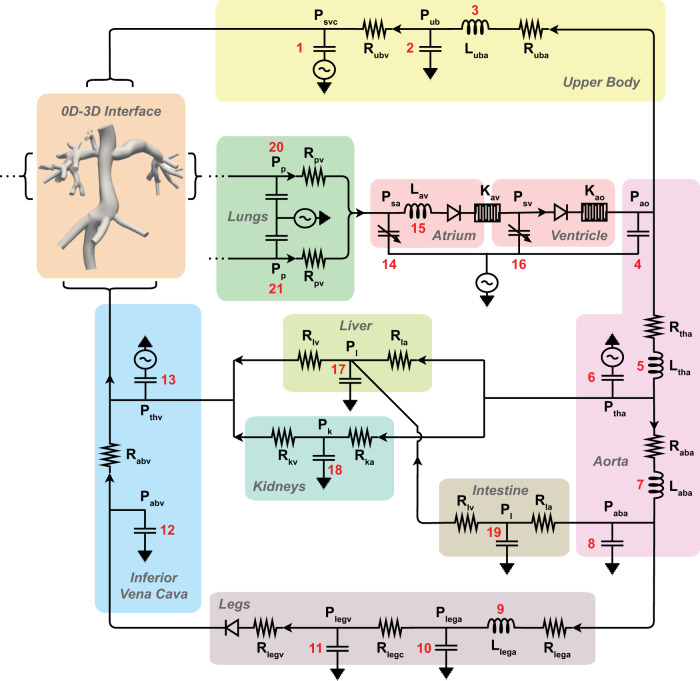
Diagram of the lumped parameter network (LPN). Connections between the 0D-3D interface, resistors (*R*), capacitors (*C*), pressures (*P*), and inductor s(*L*) components are shown.

Simulations were completed using the open-source code SimVascular to solve the time-dependent incompressible Navier–Stokes equations where blood was modeled as an incompressible Newtonian fluid, with density 1.06 g/cm^3^ and viscosity 0.04 dyn/cm^2^. For fluid–structure interaction, we employed the coupled momentum method^50^. Using the flow and deformation measurements from PC-MRI data and estimated pressure outlet boundary conditions, the stiffness (i.e., elastic modulus) of the graft was first tuned to match the observed PC-MRI luminal fractional area change over a cardiac cycle (Table 1). Then, open-loop boundary conditions were replaced with boundary conditions defined by a LPN based on previous work (Fig. 9)^51,52^. We adapted the LPN to model pediatric flows and responses to metabolic rate and further tuned the parameters to match patient-specific clinical values at each time point (Supplementary Tables 1 and 2). To account for the reduced change in total vascular resistance observed in patients during exercise compared to that modeled by Kung et al., the relationship between TVR and TVR_MET_ was defined as

1 $${{\rm{TVR}}}_{{\rm{MET}}}=\frac{-6.25* {\rm{ln}}({\rm{MET}})}{{\rm{BSA}}}+{{\rm{TVR}}}_{{\rm{MET}}\;1}.$$

In stenosis simulations, maximum aortic pressure was matched to the TEVG reference geometry by scaling the total excess blood volume, as an approximation to the body’s long-term response mechanism to decreased preload. Using increased blood volume as the main response to stenosis is supported by the finding that increases in blood volumes is one of the most significant changes in physiology that persists long term after constriction of the major venous vessels^53^.

### ePTFE graft simulations

This pipeline was repeated with models modified to mimic 16 mm diameter ePTFE graft geometry and material properties to facilitate comparison between TEVG vs ePTFE grafts.

### Calculation of oxygenation metrics

To determine oxygenation, we estimated OER and sVO_2_ using Eqs. (2) and (3), respectively. Arterial oxygen saturation and partial pressures of oxygen were estimated from literature values in Fontan patients and hemaglobin concentration was estimated from our pediatric Fontan exercise cohort^54–56^. For the equations,

2 $${\rm{OER}}=\frac{{\rm{MET}}\times 6.5\times {\rm{weight}}}{((13.26\times 1.34\times 0.94)+0.003\times {{\rm{PO}}}_{2{\rm{arterial}}})\times 10\times {\rm{CO}}}$$

and

3 $${{\rm{sVO}}}_{2}=0.94-\frac{(\frac{{\rm{MET}}\times 6.5\times {\rm{weight}}}{\rm{CO}}+0.03({{\rm{PO}}}_{2{\rm{arterial}}}-{{\rm{PO}}}_{2{\rm{venous}}}))}{13.26\times 1.34\times 10},$$

MET is the youth metabolic equivalent, PO_2arterial_ is the atrial partial pressure of oxygen (90 mmHg), PO_2venous_ is the venous partial pressure of oxygen (40 mmHg), and CO is the cardiac output. Both equations were derived using Fick’s principle.

### Calculation of ventricular metrics

Ventricular metrics were calculated directly from the simulation results. Pressure values were averaged across a complete respiratory cycle. CI was calculated as the average CI across a respiratory cycle.

4 $${\rm{CI}}=\frac{{\rm{SV}}\times {\rm{HR}}}{{\rm{BSA}}},$$

where SV is stroke volume, HR is heart rate, and BSA is patient-specific body surface area.

### Calculation of local hemodynamics

Energy efficiency was calculated across each model as

5 $$E=\frac{\mathop{\sum }\nolimits_{i = 1}^{{N}_{{\rm{out}}}}{\int}_{{A}_{i}}(p+\frac{1}{2}\rho {u}^{2}){\bf{u}}\cdot {\rm{d}}A}{\mathop{\sum }\nolimits_{i = 1}^{{N}_{{\rm{in}}}}{\int}_{{A}_{i}}(p+\frac{1}{2}\rho {u}^{2}){\bf{u}}\cdot {\rm{d}}A},$$

where *A* is the control surface, *u* is the velocity, **u** is the velocity in the normal direction to the control surface, *p* is the static pressure, and *ρ* is the fluid density. This ratio represents the percentage of energy retained by the model over one respiratory cycle.

TAWSS was computed as

6 $${\rm{TAWSS}}=\frac{\mathop{\int}\nolimits_{0}^{T}| | \overrightarrow{{\rm{WSS}}}| | {\rm{d}}t}{T},$$

where $\overrightarrow{{\rm{WSS}}}$ is the wall shear stress (the component of the traction vector on the luminal surface in the primary direction of flow) produced by blood moving across the endothelial surface and *T* is the duration of one respiratory cycle. TAWSS was then spatially averaged over areas of interest.

OSI was calculated as

7 $${\rm{OSI}}=\frac{1}{2}\left(1-\frac{| \mathop{\int}\nolimits_{0}^{T}\overrightarrow{{\rm{WSS}}}{\rm{d}}t| }{\mathop{\int}\nolimits_{0}^{T}| \overrightarrow{{\rm{WSS}}}| {\rm{d}}t}\right)$$

at each point in the model and averaged over the graft section of the model.

The first invariant of the Green strain was calculated as

8 $${I}_{E}={\rm{tr}}\left(\frac{1}{2}\left({{\bf{F}}}^{T}{\bf{F}}-{\bf{I}}\right)\right),$$

where **F** is the deformation gradient tensor, **I** is an identity tensor, and tr is the trace operator. The first invariant of the Green strain was time-averaged for magnitude, then spatially averaged across the graft section.

Non-discrete RT computation was used to calculate average RT inside the graft volume^57^. The average maximum RT is defined as

9 $${{\rm{RT}}}_{{\rm{max}}}={{\rm{max}}}_{{{\Omega }}}\left(\frac{1}{T}\mathop{\int}\nolimits_{(n-1)T}^{nT}\tau (x,t){\rm{d}}t\right)$$

where the number of simulated cycles, *n*, is selected such that the transient part of the solution is damped, *τ* is the time a fluid particle has been in the region of interest, Ω is the region of interest, *V* is volume, and *t* is a periodic function of time.

### Reporting summary

Further information on research design is available in the Nature Research Reporting Summary linked to this article.

## Supplementary information

Supplemental Material

Reporting Summary

## Data Availability

Imaging data analyzed in this study are available from the corresponding author on reasonable request and following establishment of appropriate institutional agreements.

## Code availability

All models and simulations were created in the open-source code SimVascular (simvascular.org)^49^.
