# Publisher Correction: Hemodynamic performance of tissue-engineered vascular grafts in Fontan patients

**DOI:** 10.1038/s41536-021-00157-9

**Published:** 2021-08-12

**Authors:** Erica L. Schwarz, John M. Kelly, Kevin M. Blum, Kan N. Hor, Andrew R. Yates, Jacob C. Zbinden, Aekaansh Verma, Stephanie E. Lindsey, Abhay B. Ramachandra, Jason M. Szafron, Jay D. Humphrey, Toshiharu Shin’oka, Alison L. Marsden, Christopher K. Breuer

**Affiliations:** 1 Department of Bioengineering, Stanford University, Stanford, CA USA; 2 Center for Regenerative Medicine, Abigail Wexner Research Institute at Nationwide Children’s Hospital, Columbus, OH USA; 3 The Heart Center, Nationwide Children’s Hospital, Columbus, OH USA; 4 Department of Biomedical Engineering, The Ohio State University, Columbus, OH USA; 5 Department of Pediatrics, The Ohio State University College of Medicine, Columbus, OH USA; 6 Department of Pediatrics, Stanford University, Stanford, CA USA; 7 Department of Biomedical Engineering, Yale University, New Haven, CT USA; 8 Department of Cardiothoracic Surgery, Nationwide Children’s Hospital, Columbus, OH USA; 9 Department of Surgery, The Ohio State University Wexner Medical Center, Columbus, OH USA; 10 Department of Surgery, Nationwide Children’s Hospital, Columbus, OH USA

**Keywords:** Tissue engineering, Circulation, Translational research

Correction to: *npj Regenerative Medicine* 10.1038/s41536-021-00148-w, published online 22 July 2021

The original version of this Article omitted that Erica L. Schwarz and John M. Kelly jointly led this research as equally contributing first authors. This has now been corrected in both the PDF and HTML versions of the Article.

