# Supplementary material for: Hemodynamic performance of tissue-engineered vascular grafts in Fontan patients
Source: NPJ Regen Med. 2021 Jul 22;6:38. doi: 10.1038/s41536-021-00148-w (PMC8298568; doi:10.1038/s41536-021-00148-w)
Supplement: Supplementary file 1 — Supplemental Material [file 41536_2021_148_MOESM1_ESM.pdf]

Supplemental Information

Supplementary Figure 1 - Location of vessel for deformation assesment
Supplementary Figure 2 - Comparison of TEVG and Glenn growth
Supplementary Figure 3 - Evaluation of cross sectional area changes
Supplementary Figure 4 - Additional validation against echocardiograph data
Supplementary Figure 5 - First invariant of Green strain data
Supplementary Figure 6 - Examples of local hemodynamic results
Supplementary Table 1 - Target tuning values and tuned parameters
Supplementary Table 2 - Tuned initial conditions

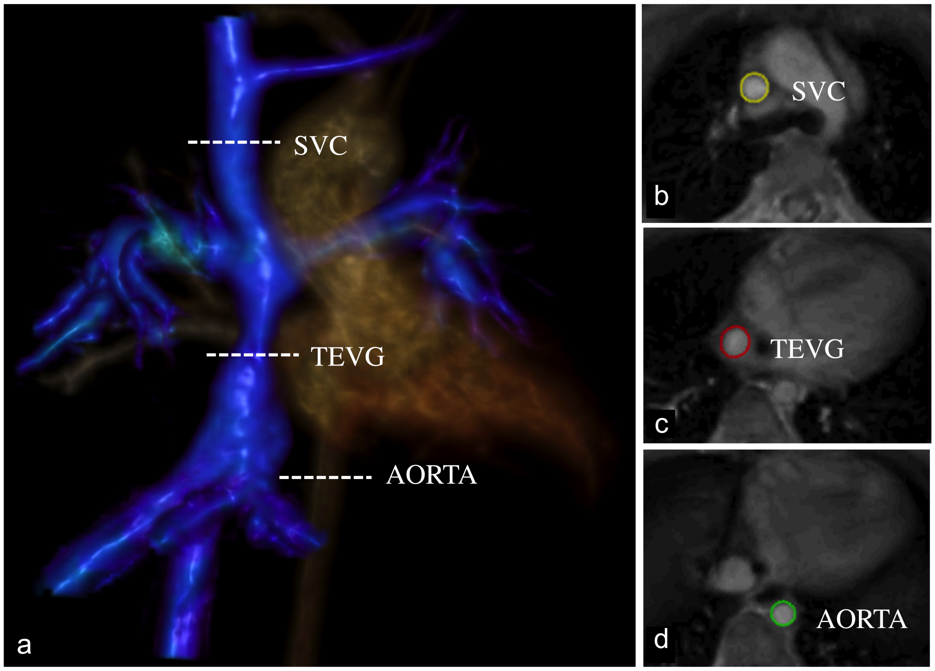

Supplementary Figure 1: Location of Vessel for Deformation Assessment: (a) Location of SVC (mid-way between innominate vein and branch pulmonary arteries), TEVG (mid-way between branch pulmonary arteries and hepatic veins and intrahepatic inferior vena cava junction) and Aorta (Descending Aorta just above diaphragm). (b-d) Cross-section of SVC, TEVG and Aorta from (a) for dynamic deformation assessment from cine images tracking the vessels throughout the cardiac cycle (change in size). (SVC = Superior Vena Cava, TEVG = Tissue-engineered Vascular Graft)

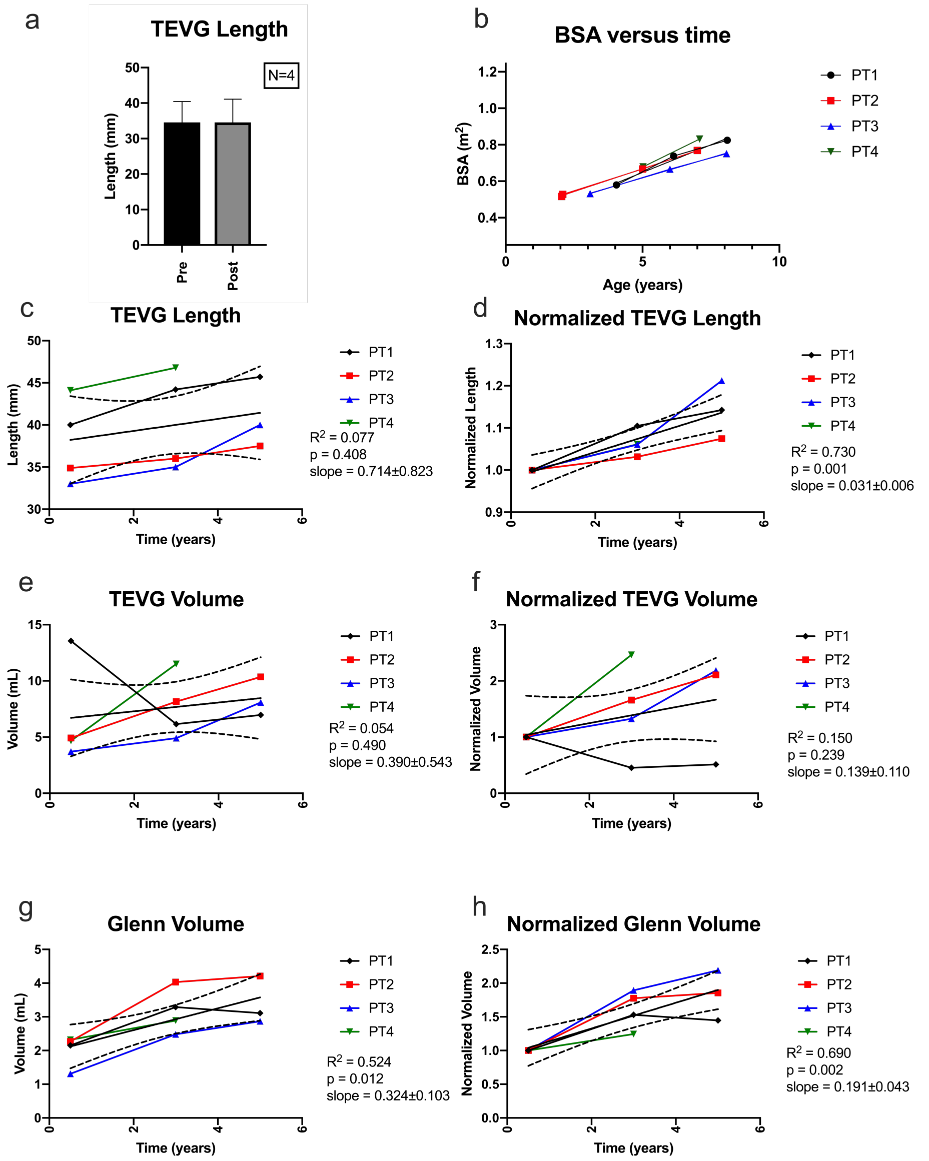

Supplementary Figure 2: Comparison of TEVG and Glenn growth for the entire patient cohort (a) TEVG length measured angiographically pre and post balloon angioplasty of the TEVG. (b) Linear increase in BSA for all patients. (c) Absolute values of TEVG length. (d) TEVG length normalized to the length at the 6 month time point. (e) Absolute values of TEVG volume. (f) TEVG volume normalized to the volume at the 6 month time point. (g) Absolute values of Glenn volume. (h) Glenn volume normalized to the volume at the 6 month time point. (BSA = body surface area, TEVG = Tissue-Engineered Vascular Graft, Glenn = superior vena cava to pulmonary artery connection)

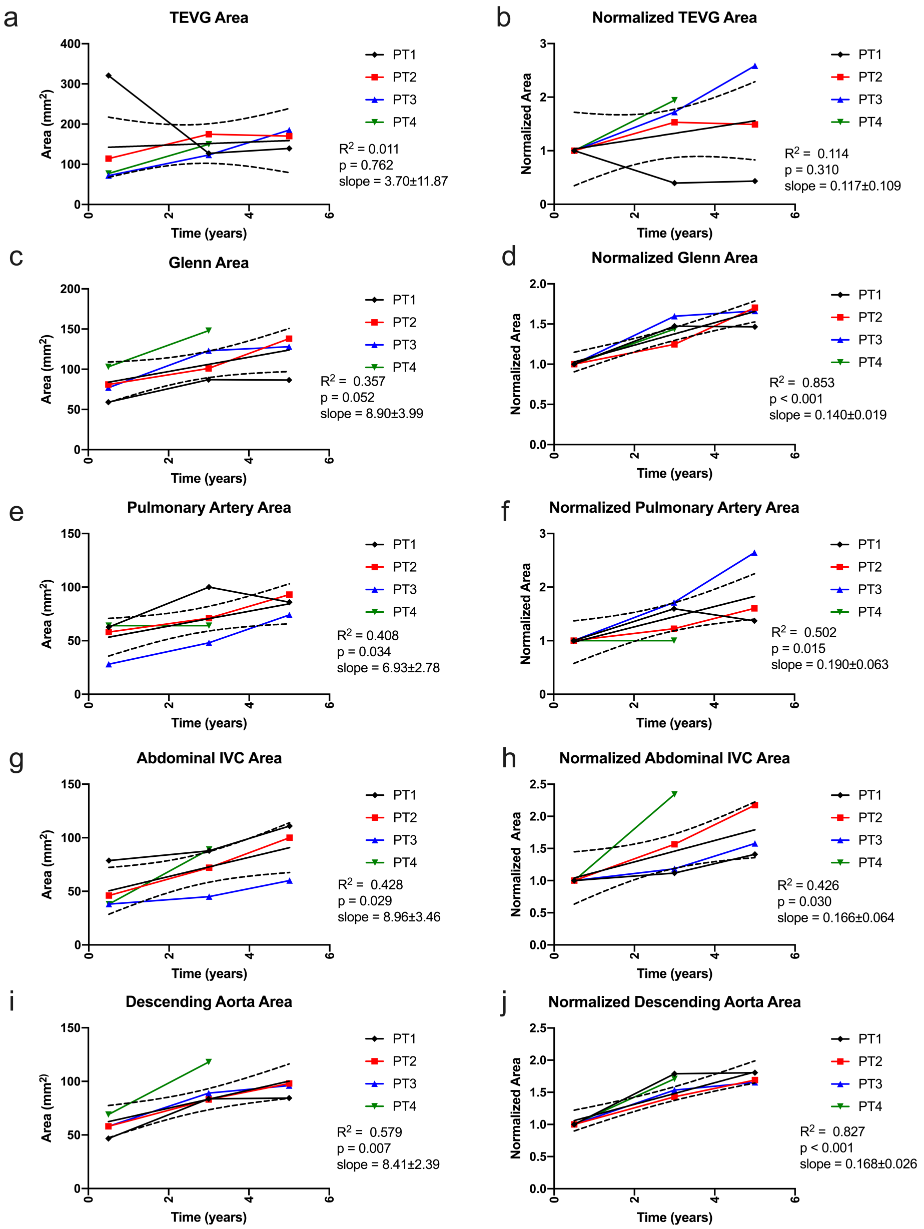

Supplementary Figure 3: Evaluation of cross sectional area changes of the TEVG and native vasculature. (a) TEVG area. (b) TEVG area normalized to the 6 month time point. (c) Glenn area. (d) Glenn area normalized to the 6 month time point. (e) Pulmonary artery area. (f) Pulmonary artery area normalized to the 6 month time point. (g) Abdominal IVC area. (h) Abdominal IVC area normalized to the 6 month time point. (i) Descending aorta area. (j) Descending aorta normalized to the 6 month time point. (TEVG = Tissue-Engineered Vascular Graft, Glenn = superior vena cava to pulmonary artery connection)

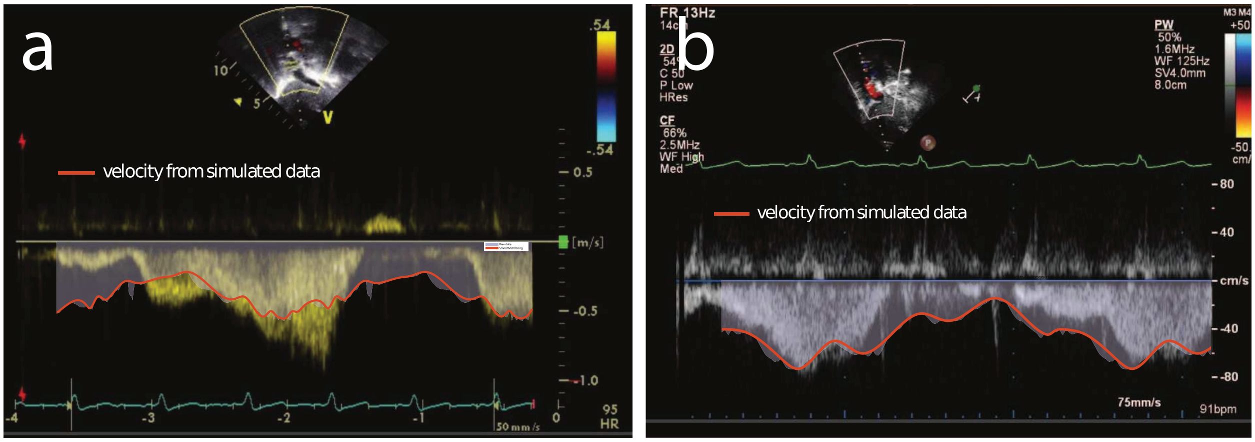

Supplementary Figure 4: Validation against echocardiograph data. (a) Patient 1 at 2 years post-implantation (16 mm diameter, echocardiogram) velocity compared to Patient 1 at 6 months post-implantation (18 mm diameter, simulated from MRI data) velocity. (b) Patient 1 at 3 years post-implantation (16 mm diameter, echocardiogram) velocity compared to Patient 1 at 3 years post-implantation (18 mm diameter, simulated from MRI data) velocity.

Additional local hemodynamics

The first invariant of the Green strain was comparable across all TEVG models (Supplementary Figure 5). The stiffness of the ePTFE graft models and the reference geometry model with rigid material properties maintained the strain measure at close to zero. The graft-averaged first invariant of the Green strain remained constant at all diameter levels, although at high stenosis levels, an increase in the first invariant of the Green strain was observable proximal to the narrowing.

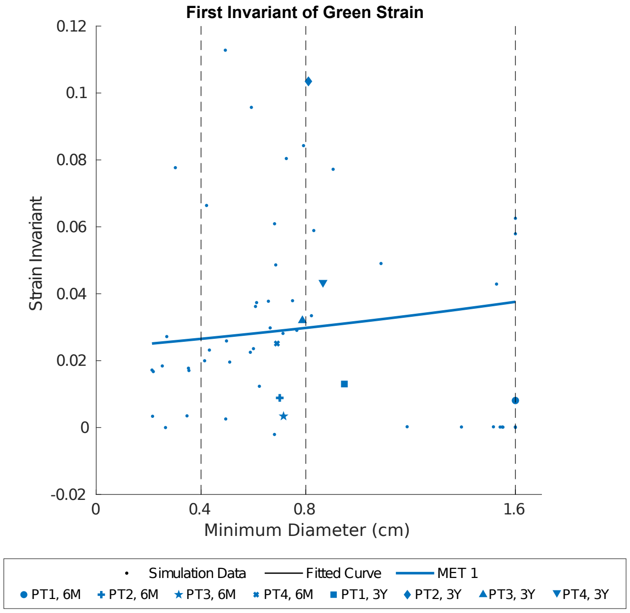

Supplementary Figure 5: Average first invariant of Green strain of the Fontan graft at MET1 across one respiratory cycle.

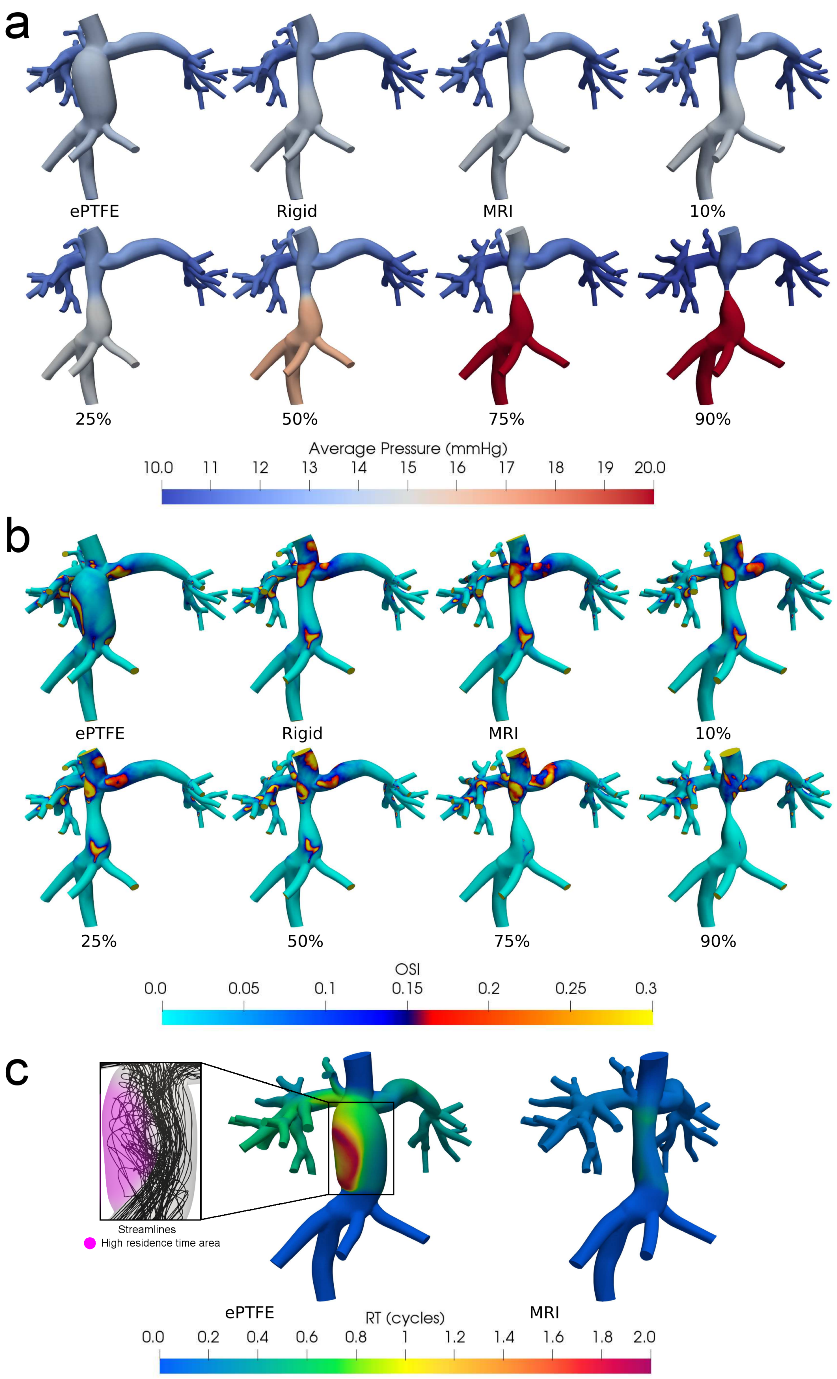

Supplementary Figure 6: Examples of local hemodynamic results for Patient 3 at 6 months post-implantation. (a) Pressure results for models. (b) WSS results for models. (c) RT results for models.

	**Patient 1**		**Patient 2**		**Patient 3**		**Patient 4**	
**Months post-implantation**	6	36	6	36	6	36	6	36
Capacitances (ml mmHg^{-1})								
Caba	2.28E-01	2.53E-01	1.50E-01	1.51E-01	2.16E-01	3.02E-01	1.86E-01	3.88E-01
Cabv	4.62E+00	5.12E+00	3.05E+00	3.05E+00	4.38E+00	6.12E+00	3.77E+00	7.86E+00
Cao	3.59E-01	3.98E-01	2.37E-01	2.37E-01	3.41E-01	4.76E-01	2.93E-01	6.11E-01
Ci	7.73E-01	8.57E-01	5.10E-01	5.11E-01	7.33E-01	1.02E+00	6.31E-01	1.32E+00
Ck	1.29E+00	1.43E+00	8.53E-01	8.55E-01	1.23E+00	1.71E+00	1.06E+00	2.20E+00
Cl	3.11E+00	3.44E+00	2.05E+00	2.05E+00	2.94E+00	4.12E+00	2.53E+00	5.29E+00
Clega	1.01E+00	1.12E+00	6.65E-01	6.66E-01	9.55E-01	1.34E+00	8.22E-01	1.72E+00
Clegv	5.13E+00	5.69E+00	3.38E+00	3.39E+00	4.87E+00	6.80E+00	4.19E+00	8.74E+00
Cp	3.32E+00	3.84E+00	3.88E+00	7.89E+00	5.43E+00	6.34E+00	1.15E+00	5.66E+00
Csvc	1.03E+00	1.14E+00	6.77E-01	6.78E-01	9.73E-01	1.36E+00	8.38E-01	1.75E+00
Cthao	1.08E-01	1.19E-01	7.10E-02	7.12E-02	1.02E-01	1.43E-01	8.79E-02	1.83E-01
Cthv	5.13E-01	5.69E-01	3.38E-01	3.39E-01	4.87E-01	6.80E-01	4.19E-01	8.74E-01
Cub	2.21E+00	2.45E+00	1.46E+00	1.46E+00	2.09E+00	2.92E+00	1.80E+00	3.76E+00
Inductances (mm Hg s^{2} ml^{-1})								
Laba	1.33E-03	1.33E-03	1.33E-03	1.33E-03	1.33E-03	1.33E-03	1.33E-03	1.33E-03
Lav	1.70E-05	2.21E-06	1.81E-06	7.66E-08	1.13E-05	5.87E-07	8.35E-07	1.03E-07
Llega	1.33E-03	1.33E-03	1.33E-03	1.33E-03	1.33E-03	1.33E-03	1.33E-03	1.33E-03
Ltha	1.33E-03	1.33E-03	1.33E-03	1.33E-03	1.33E-03	1.33E-03	1.33E-03	1.33E-03
Luba	5.48E-04	5.48E-04	5.48E-04	5.48E-04	5.48E-04	5.48E-04	5.48E-04	5.48E-04
Resistances (mm Hg s ml^{-1})								
Rabv	8.25E-02	7.63E-02	1.13E-01	1.13E-01	8.58E-02	6.68E-02	9.61E-02	5.53E-02
Raba	8.53E-01	7.90E-01	1.17E+00	1.16E+00	8.88E-01	6.91E-01	9.94E-01	5.73E-01
Ria	1.82E+01	1.69E+01	2.49E+01	2.49E+01	1.90E+01	1.48E+01	2.12E+01	1.22E+01
Riv	3.31E-01	3.07E-01	4.53E-01	4.52E-01	3.45E-01	2.68E-01	3.86E-01	2.22E-01
Rka	8.31E+00	7.69E+00	1.14E+01	1.13E+01	8.65E+00	6.73E+00	9.68E+00	5.58E+00
Rkv	7.79E-01	7.21E-01	1.06E+00	1.06E+00	8.10E-01	6.30E-01	9.07E-01	5.22E-01
Rla	1.18E+01	1.09E+01	1.61E+01	1.61E+01	1.23E+01	9.55E+00	1.37E+01	7.92E+00
Rlega	1.12E+00	1.04E+00	1.54E+00	1.53E+00	1.17E+00	9.10E-01	1.31E+00	7.54E-01
Rlegc	2.66E+00	2.46E+00	3.63E+00	3.63E+00	2.77E+00	2.15E+00	3.10E+00	1.78E+00
Rlegv	6.19E-01	5.73E-01	8.46E-01	8.45E-01	6.45E-01	5.01E-01	7.22E-01	4.16E-01
Rlv	7.39E-02	6.84E-02	1.01E-01	1.01E-01	7.70E-02	5.99E-02	8.61E-02	4.96E-02
Rpv	2.99E-02	4.63E-02	1.16E-03	5.93E-02	6.59E-03	9.24E-03	1.11E-01	1.58E-02
Rsvc	8.25E-02	7.63E-02	1.13E-01	1.13E-01	8.58E-02	6.68E-02	9.61E-02	5.53E-02
Rtha	1.12E-01	1.04E-01	1.54E-01	1.53E-01	1.17E-01	9.11E-02	1.31E-01	7.55E-02
Ruba	9.44E-01	8.74E-01	1.29E+00	1.29E+00	9.83E-01	7.65E-01	1.10E+00	6.34E-01
Rubv	3.30E+00	3.05E+00	4.51E+00	4.50E+00	3.43E+00	2.67E+00	3.84E+00	2.21E+00
Heart Parameters								
Csa (ml mm Hg^{-1})	4.99E-02	6.64E+00	1.78E+00	6.81E-01	3.18E+00	3.79E+00	5.40E+00	7.65E-01
Psar (mm Hg)	3.34E-02	1.87E-04	2.10E-01	1.86E-01	1.25E-02	3.93E-03	3.42E-03	3.40E-01
Dsa (ml^{-1})	3.48E-01	1.22E-01	1.09E-01	2.09E-01	1.52E-01	2.34E-01	1.40E-01	2.82E-01
Kav (mm Hg s^{2} ml^{-2})	2.25E-11	4.80E-05	1.49E-08	7.90E-07	1.05E-05	5.80E-06	1.31E-05	1.61E-06
Kao (mm Hg s^{2} ml^{-2})	7.39E-06	1.90E-09	3.11E-06	2.87E-07	4.75E-09	1.14E-06	2.68E-07	2.45E-05
Rmyo (mm Hg s ml^{-1})	1.25E-04	1.02E-03	2.01E-04	1.86E-03	1.09E-06	2.34E-12	1.89E-03	2.50E-03
Vsvo (ml)	-4.15E+01	-3.03E+01	-6.45E+01	-5.38E+01	-5.39E+01	-5.06E+01	-3.94E+01	-4.34E+01
Vsao (ml)	1.50E+01	-6.38E+01	-1.08E+01	4.48E+00	-8.29E+00	-1.71E+01	-3.65E+01	5.50E+00
Tuning Values								
Heart rate (beats/min)	138.24	122.70	140.52	107.02	131.58	90.35	100.84	79.05
Mean mid-TEVG pressure (mmHg)	13	“	“	“	“	“	“	“
Mid-TEVG pressure pulse (mmHg)	1-2	“	“	“	“	“	“	“
Pulmonary flow split (Q_L:Q_R)	32:68	33:67	53:47	53:47	45:55	45:55	46:54	45:54
Cardiac Output (L/min)	2.10	2.27	1.77	1.77	2.02	2.59	1.80	3.13
End Diastolic Ventricular Volume (ml)	43.58	50.48	36.09	45.63	36.41	45.59	46.43	56.85

Supplementary Table 1: Target tuning values and tuned parameters for each MRI geometry model at MET 1

	**Patient 1**		**Patient 2**		**Patient 3**		**Patient 4**	
**Months post-implantation**	6	36	6	36	6	36	6	36
Initial condition								
1	1.17E+01	1.42E+01	1.53E+01	1.57E+01	1.45E+01	1.31E+01	1.48E+01	1.45E+01
2	5.83E+01	5.89E+01	6.05E+01	6.77E+01	5.38E+01	5.29E+01	5.56E+01	5.94E+01
3	2.78E+00	7.63E+00	4.19E+00	2.86E+00	7.00E+00	3.56E+00	1.57E+00	1.82E+00
4	6.09E+01	6.55E+01	6.59E+01	7.14E+01	6.06E+01	5.56E+01	5.73E+01	6.06E+01
5	1.05E+01	1.19E+01	7.84E+00	8.79E+00	9.14E+00	1.06E+01	7.64E+00	1.46E+01
6	5.97E+01	6.43E+01	6.47E+01	7.00E+01	5.96E+01	5.47E+01	5.63E+01	5.95E+01
7	5.32E+00	6.95E+00	4.19E+00	4.34E+00	5.47E+00	4.96E+00	3.37E+00	6.39E+00
8	5.52E+01	5.89E+01	5.98E+01	6.50E+01	5.48E+01	5.13E+01	5.30E+01	5.58E+01
9	8.05E+00	1.10E+01	6.93E+00	7.28E+00	8.57E+00	8.70E+00	5.86E+00	1.07E+01
10	4.62E+01	4.75E+01	4.92E+01	5.39E+01	4.48E+01	4.34E+01	4.53E+01	4.78E+01
11	1.94E+01	2.05E+01	2.18E+01	2.21E+01	2.16E+01	1.97E+01	2.05E+01	2.05E+01
12	1.33E+01	1.44E+01	1.51E+01	1.54E+01	1.55E+01	1.44E+01	1.49E+01	1.47E+01
13	1.26E+01	1.41E+01	1.48E+01	1.51E+01	1.51E+01	1.39E+01	1.43E+01	1.41E+01
14	1.58E+01	1.55E+01	2.09E+01	1.51E+01	1.53E+01	1.52E+01	1.66E+01	1.53E+01
15	0.00E+00	0.00E+00	0.00E+00	0.00E+00	0.00E+00	0.00E+00	0.00E+00	0.00E+00
16	4.19E+01	5.38E+01	3.57E+01	4.55E+01	3.67E+01	4.11E+01	4.56E+01	5.54E+01
17	1.30E+01	1.42E+01	1.48E+01	1.52E+01	1.53E+01	1.42E+01	1.46E+01	1.44E+01
18	1.71E+01	1.82E+01	1.88E+01	1.95E+01	1.87E+01	1.76E+01	1.81E+01	1.83E+01
19	1.38E+01	1.47E+01	1.52E+01	1.57E+01	1.57E+01	1.47E+01	1.51E+01	1.51E+01
20	8.29E+00	1.37E+01	1.24E+01	1.40E+01	1.25E+01	1.19E+01	1.29E+01	1.31E+01
21	8.29E+00	1.36E+01	1.24E+01	1.42E+01	1.26E+01	1.19E+01	1.28E+01	1.33E+01

Supplementary Table 2: Tuned initial conditions for each MRI geometry model at MET 1 corresponding to the labeled LPN in Figure 9
