# Supplementary material for: Hemodynamic performance of tissue-engineered vascular grafts in Fontan patients
Source: NPJ Regen Med. 2021 Jul 22;6:38. doi: 10.1038/s41536-021-00148-w (PMC8298568; doi:10.1038/s41536-021-00148-w)
Supplement: Supplementary file 2 — Reporting Summary [file 41536_2021_148_MOESM2_ESM.pdf]

nature research

Corresponding author(s): Erica L Schwarz
John M Kelly

Last updated by author(s): Mar 2, 2021

Reporting Summary

Nature Research wishes to improve the reproducibility of the work that we publish. This form provides structure for consistency and transparency in reporting. For further information on Nature Research policies, see our Editorial Policies and the Editorial Policy Checklist.

Statistics

For all statistical analyses, confirm that the following items are present in the figure legend, table legend, main text, or Methods section.

n/a	Confirmed	
☐	☒	The exact sample size (n) for each experimental group/condition, given as a discrete number and unit of measurement
☐	☒	A statement on whether measurements were taken from distinct samples or whether the same sample was measured repeatedly
☐	☒	The statistical test(s) used AND whether they are one- or two-sided<br>*Only common tests should be described solely by name; describe more complex techniques in the Methods section.*
☐	☒	A description of all covariates tested
☐	☒	A description of any assumptions or corrections, such as tests of normality and adjustment for multiple comparisons
☐	☒	A full description of the statistical parameters including central tendency (e.g. means) or other basic estimates (e.g. regression coefficient) AND variation (e.g. standard deviation) or associated estimates of uncertainty (e.g. confidence intervals)
☐	☒	For null hypothesis testing, the test statistic (e.g. F, t, r) with confidence intervals, effect sizes, degrees of freedom and P value noted<br>*Give P values as exact values whenever suitable.*
☐	☒	For Bayesian analysis, information on the choice of priors and Markov chain Monte Carlo settings
☐	☒	For hierarchical and complex designs, identification of the appropriate level for tests and full reporting of outcomes
☐	☒	Estimates of effect sizes (e.g. Cohen's d, Pearson's r), indicating how they were calculated

Our web collection on statistics for biologists contains articles on many of the points above.

Software and code

Policy information about availability of computer code

Data collection	The open-source SimVascular (simvascular.org) was used to create physiological models and simulations.
Data analysis	*Provide a description of all commercial, open source and custom code used to analyse the data in this study, specifying the version used OR state that no software was used.*

For manuscripts utilizing custom algorithms or software that are central to the research but not yet described in published literature, software must be made available to editors and reviewers. We strongly encourage code deposition in a community repository (e.g. GitHub). See the Nature Research guidelines for submitting code & software for further information.

Data

Policy information about availability of data

All manuscripts must include a data availability statement. This statement should provide the following information, where applicable:

- Accession codes, unique identifiers, or web links for publicly available datasets
- A list of figures that have associated raw data
- A description of any restrictions on data availability

The details of the clinical trial can be accessed at clinicaltrials.gov.

Field-specific reporting

Please select the one below that is the best fit for your research. If you are not sure, read the appropriate sections before making your selection.

☒ Life sciences ☐ Behavioural & social sciences ☐ Ecological, evolutionary & environmental sciences

For a reference copy of the document with all sections, see nature.com/documents/nr-reporting-summary-flat.pdf

Life sciences study design

All studies must disclose on these points even when the disclosure is negative.

Sample size	We initially planned on enrolling a total of 6 patients. Since we experienced a higher incidence of stenosis than expected, we concluded at 4 patients total. Since this was a pilot study to look at rates of adverse events among patients who receive these vascular grafts, the study was not powered for hypothesis testing
Data exclusions	We conducted analysis of the growth of the tissue engineered vascular graft including all patients and a subanalysis was additionally undertaken excluding one patient as the construction of their vascular graft was significantly different then the other study participates and may have affected the time course of remodeling of the vascular graft. We attempted to clearly state in the body of the manuscript when data is presented with the exclusion of this patient.
Replication	Measurements from the clinical data were analyzed by two clinicians and results compared between observers to determine the accuracy of the measurements.
Randomization	Study participants were not randomly allocated to treatment as there was only a single interventional arm to the study. Covariates such as age, gender and cardiac lesion were not controlled for as inclusion criteria included patients undergoing Fontan completion per current standard of care with surgery typically undertaken at 2-4 years of age following the Glenn procedure.
Blinding	Study blinding was not relevant as all study participants were allocated for treatment with placement of a tissue engineered vascular graft. Initial pilot experiment in a patient population with orphan designation thus limiting our ability to randomize and blind.

Reporting for specific materials, systems and methods

We require information from authors about some types of materials, experimental systems and methods used in many studies. Here, indicate whether each material, system or method listed is relevant to your study. If you are not sure if a list item applies to your research, read the appropriate section before selecting a response.

Materials & experimental systems

n/a	Involved in the study	
☒	☐	Antibodies
☒	☐	Eukaryotic cell lines
☒	☐	Palaeontology and archaeology
☒	☐	Animals and other organisms
☐	☒	Human research participants
☐	☒	Clinical data
☒	☐	Dual use research of concern

Methods

n/a	Involved in the study	
☒	☐	ChIP-seq
☒	☐	Flow cytometry
☒	☐	MRI-based neuroimaging

Human research participants

Policy information about studies involving human research participants

Population characteristics	Covariant-relevant patient characteristics are summarized in the manuscript. Patients aged between 2-4 years old, 1 male patient and 3 female patients. All study participants have single ventricle physiology with 2 patients having hypoplastic left heart syndrome, 1 patient with unbalanced atrioventricular septal defect with pulmonary atresia and 1 patient with pulmonary atresia with an intact ventricular septum and hypoplastic right ventricle.
Recruitment	All patients who were considered candidates to undergo Extracardiac total cavopulmonary connection (EC TCPC) for completion of a modified Fontan for palliation of their congenital cardiac anomaly during the course of the investigation at our institution were considered for enrollment in the trial. No patient was excluded based on age, gender, or ethnicity. Inclusion criteria included patients with single ventricle anomalies who were candidates for extracardiac total cavopulmonary connection, volunteered, and provided informed consent
Ethics oversight	Institutional review board (IRB) approval was granted by Yale University (Human Investigation Committee #0701002198) and Nationwide Children’s Hospital (IRB12-00357 and IRB15-00013).

Note that full information on the approval of the study protocol must also be provided in the manuscript.

Clinical data

Policy information about clinical studies

All manuscripts should comply with the ICMJE guidelines for publication of clinical research and a completed CONSORT checklist must be included with all submissions.

Clinical trial registration	FDA-approved clinical trial (IDE 14127)
Study protocol	Study protocol can be accessed on clinicaltrials.gov
Data collection	We received FDA approval in December 2009 while at Yale New Haven Children's Hospital associated with Yale University. We implanted our first patient in August 2011. We relocated the study to Nationwide Children's Hospital associated with The Ohio State University in September 2012. After completion of required inspection of the institutional facility we resumed enrollment in March 2014 with a completion of all clinical follow-ups in August 2017. Subsequently patients were enrolled in an observational study to evaluate longterm graft related complications and assess growth. This manuscript details the results of the initial clinical trial and subsequent observational study.
Outcomes	Primary end points of the study included determination of graft failure rates and graft related morbidity and mortality. Graft failure was defined as any graft narrowing/occlusion or dilation/rupture requiring surgical or endovascular graft replacement. Graft related morbidity and mortality included any post-operative complication such as any thromboembolic or infectious event that required treatment and was thought likely to be caused by the tissue engineered vascular graft as determined by the investigators and confirmed by the data safety monitoring board
